# Targeting Vascular Endothelial Growth Factor Receptors as a Therapeutic Strategy for Osteoarthritis and Associated Pain

**DOI:** 10.7150/ijbs.79125

**Published:** 2023-01-01

**Authors:** Kaige Ma, Gurjit Singh, Jun Wang, InSug O-Sullivan, Gina Votta-Velis, Benjamin Bruce, Arivarasu Natarajan Anbazhagan, Andre J. van Wijnen, Hee-Jeong Im

**Affiliations:** 1 Department of Biomedical Engineering, the University of Illinois at Chicago, Chicago, IL, USA.; 2 Department of Orthopaedics, Union Hospital, Tongji Medical College, Huazhong University of Science and Technology, Wuhan, China.; 3 Department of Anesthesiology, the University of Illinois at Chicago, Chicago, IL, USA.; 4 Jesse Brown Veterans Affairs Medical Center (JBVAMC) at Chicago, IL 60612, USA.; 5 Department of Medicine, the University of Illinois at Chicago, Chicago, IL, USA.; 6 Department of Biochemistry, University of Vermont, Burlington, VT 05405, USA.

**Keywords:** Osteoarthritis, cartilage degeneration, joint pain, VEGFRs, pazopanib

## Abstract

Pain is the major reason that patients suffering from osteoarthritis (OA) seek medical care. We found that vascular endothelial growth factors (VEGFs) mediate signaling in OA pain pathways. To determine the specific contributions of VEGFs and their receptors (VEGFRs) to joint pathology and pain transmission during OA progression, we studied intra-articular (IA) injections of VEGF ligands into murine knee joints. Only VEGF ligands specific for the activation of VEGFR1, but not VEGFR2, induced allodynia within 30 min. Interventions in OA by inhibitors of VEGFRs were done *in vivo* using a preclinical murine OA model by IA injections of selective inhibitors of VEGFR1/VEGFR2 kinase (pazopanib) or VEGFR2 kinase (vandetanib). OA phenotypes were evaluated using pain-associated murine behavioral tests and histopathologic analyses. Alterations in VEGF/VEGFR signaling by drugs were determined in knee joints, dorsal root ganglia, and spinal cord by immunofluorescence microscopy. Pazopanib immediately relieved OA pain by interfering with pain transmission pathways. Pain reduction by vandetanib was mainly due to the inhibition of cartilage degeneration by suppressing VEGFR2 expression. In conclusion, IA administration of pazopanib, which simultaneously inhibits VEGFR1 and VEGFR2, can be developed as an ideal OA disease-modifying drug that rapidly reduces joint pain and simultaneously inhibits cartilage degeneration.

## Introduction

Osteoarthritis (OA) pain is a major reason that patients seek medical care. The etiology of pain in OA, however, remains unclear. Currently, there is no OA-disease-modifying drug (OADMD) that can reverse or prevent OA progression 1-4. A better understanding of the molecular mechanisms driving the pathology of OA will be essential to identify new and effective treatment avenues for OA.

In OA patients, increasing OA severity is directly correlated with the expression of genes encoding vascular endothelial growth factors (VEGFs) in joints 5-7. Moreover, knee joints injected with VEGF protein develop OA-like joint pathology 8, and inhibition of VEGF promotes cartilage repair by suppressing angiogenesis within the joint 6,9. However, the intricate actions of VEGF in OA progression may not be restricted to roles in angiogenesis, inflammation, or pathological changes in the integrity of cartilage in joints. Recent reports suggest that the biological roles of VEGF signaling indeed may go beyond promoting vascular epithelial growth and angiogenesis and perhaps also include a more direct role in pain transmission 10,11.

VEGF ligands interact with their corresponding receptor tyrosine kinases VEGFR1, VEGFR2 or VEGFR3 to trigger their biological functions 7. VEGFA activates VEGFR1 and VEGFR2; VEGFB and VEGF-related protein placental growth factor (PGF/PlGF) activate VEGFR1; VEGFC and VEGFD activate VEGFR1 and VEGFR3 12. A parapoxvirus-encoded VEGF member (VEGFE) exclusively activates VEGFR2 12. The redundant and compensatory roles of selective VEGFR1 ligands (VEGFA, VEGFB, PlGF) were reported 10,13,14. In treating OA, targeting the specific receptors upon which these ligands converge may be more efficacious than targeting individual ligands.

VEGFs are found in normal cartilage, but only osteoarthritic cartilage expresses VEGFRs, including VEGFR1 and VEGFR2 15,16. The roles of VEGFR1 and VEGFR2 signaling and specific isoforms involved in the disease process have not been clearly delineated. Selvaraj and colleagues 13 showed a role for VEGFR1 in cancer pain, while our group reported evidence for a role for VEGFR2 in cartilage degeneration 9. VEGFA, a ligand that activates VEGFR1 and VEGFR2, is involved in cartilage degeneration and OA pain transmission 6. Hence, VEGF/VEGFR signaling may have prominent roles in OA progression and associated pain.

The present study was performed to determine the distinct roles of VEGFR1 and VEGFR2 and whether an inhibitor targeting VEGFR1 and VEGFR2 can restore joint function in OA by **(i)** eliciting rapid relief of joint pain and **(ii)** decelerating OA disease progression.

## Materials and methods

### Experimental design

This study was performed to evaluate the contributions of VEGF and its receptors (VEGFRs) to joint pathology and pain transmission during the progression of OA. This objective was addressed by**: (i)** examining the effects of VEGFA stimulation in C28/I2 human immortalized chondrocytes *in vitro* upon siRNA silencing of* VEGFR1* or *VEGFR2*, and VEGFA stimulation in the synovium of VEGFA-induced murine knee joint pain model, **(ii)** studying the effect of intra-articular (IA) injection of selective inhibitors of VEGFR1/VEGFR2 kinase (pazopanib) or VEGFR2 kinase (vandetanib) on attenuating knee joint pain and OA progression, **(iii)** characterizing OA phenotypes using pain-associated behavioral tests and histopathological analyses, **(iv)** delineating the altered VEGF/VEGFR signaling changes in knee joints, dorsal root ganglia (DRG), and spinal cord by immunofluorescence microscopy. The investigators determined the sample size according to previous experimental experience. The exact number (n) of tissue samples or cell samples used in each experiment is indicated in the respective figure legends. For *in vivo* experiments, data from animals that died or had severe health problems during the experiments were excluded. Samples were assigned randomly to the experimental and control groups. Animal or sample allocation and data acquisition *in vivo* or *in vitro* were performed in a blinded manner. The investigators were not blinded during data analysis.

### Cell culture and siRNA transfection

We purchased a C28/I2 human immortalized chondrocyte cell line from Sigma-Aldrich Co. (St Louis*,* MO*,* USA*)*. We maintained cells (passages 3-10) in high-glucose DMEM (EMD Millipore Cat. No. SLM-120-B) supplemented with 10% fetal bovine serum (EMD Millipore Cat. No. ES-009-B) and 1% penicillin-streptomycin (Mediatech, Manassas, VA). Cells in the exponential growth phase were plated in 12-well plates. After one day of incubation, cells were transfected with a set of three siVEGFR1- or siVEGFR2-specific small interfering RNAs (siRNAs) or Trilencer-27 fluorescent-labeled transfection control siRNA duplex (OriGENE Inc, SR SR301628, SR320782, SR30002) using siTran 2.0 siRNA transfection reagent (OriGENE Inc, TT320001) for 18 h following the manufacturer's protocol, followed by either human VEGFA (100 ng/ml) or vehicle (PBS with 2% bovine serum albumin, BSA) exposure for overnight.

### Mouse maintenance and generation of the experimental mouse OA model

Male and female C57BL/6 mice were procured from the Jackson Laboratory (Bar Harbor, ME) and kept in the animal care facility of the Jesse Brown Veterans Affairs Medical Center (JBVAMC), Chicago, in a temperature-controlled room (25 ± 5 °C) with a 12 h light-dark cycle. All animal studies were duly approved by the Institutional Animal Care Committee of the University of Illinois at Chicago and the JBVMC. A schematic representation of the protocol is given in Fig. 2A.

OA was induced in 12-week-old mice by partial medial meniscectomy (PMM) as described previously by our group 10,17. Briefly, mice were pretreated with 0.1 mg*/*kg buprenorphine subcutaneously and then commonly anesthetized intraperitoneally with ketamine (100 mg/kg) and xylazine (5 mg/kg). After confirming adequate anesthesia by careful pinching tests, the left hind leg hair was shaved, and the leg was thoroughly scrubbed with a topical antiseptic solution (chlorhexidine gluconate) and draped in a sterile fashion. Animals were placed in a supine position, and an approximately 1 cm left knee incision was made with a #15 scalpel blade. The knee joint was identified from the tibia and femur, and the medial menisci-tibial ligament was identified using anatomic landmarks. To perform PMM, which destabilizes the ligaments, a micro scalpel was used at a depth of 0.5 mm to remove the meniscus at the midline. Sham surgery was performed by following the same procedures except for the meniscectomy. Suturing the skin incision was done with sterile silk braided sutures (suture size 5.0). PMM and sham surgery were performed under anesthesia and sterile conditions using a medial parapatellar approach.

### Rationale for dosing VEGFR inhibitors *in vivo*

Pazopanib, a Food and Drug Administration (FDA)-approved small-molecule inhibitor of VEGFR1 and VEGFR2^18^,^19^. Vandetanib is a selective kinase inhibitor of VEGFR2 20. To select the optimal concentration of drugs, we conducted dose-dependent pain response studies using five different concentrations of pazopanib or vandetanib (5 µL of 0.5, 1, 1.5, 2, or 3 mg/mL/knee) by IA injection into the left knee joint cavity of PMM mice at week 4 (treatment for early OA) after surgery. A pazopanib concentration of 1.5 mg/mL exerted a maximal pain-relieving effect within 30 min after IA injection, and the analgesic effect lasted for three days, and a higher concentration of pazopanib (3 mg/mL) did not further improve the pain condition (Fig. S1). There was no pain-relieving effect at any tested concentration of vandetanib we tested (data not shown). IA injection twice per week was the maximal frequency that avoided repeated IA puncture-induced joint sensitivity (using a 30-gauge needle). Based on these dose-dependence studies, we thereafter used 1.5 mg/mL concentration for IA injection of both drugs in 5 µL (5% DMSO in PBS) twice a week (15 µg/week, 0.75 mg/kg for a 20 g mouse).

### IA injection of growth factors, drugs, and vehicle

Mice were anesthetized by intraperitoneal injection with ketamine (60 mg/kg) and xylazine (3 mg/kg). After confirming adequate anesthesia, the left hind leg hair was shaved, and the leg was thoroughly scrubbed with 70% ethanol. VEGFA (10 ng/knee; 5 μL, 493-MV-025/CF, R&D system, Minneapolis, MN), VEGFB (10 ng/knee; 5 μL, 767-VE-010/CF, R&D system, Minneapolis, MN), or VEGFE (10 ng/knee; 5 μL, DA36516S, OriGene Technologies, Rockville, MD) was intraarticularly injected into the left knee joint cavity of naive mice. The identical amounts of sterile PBS containing 2% BSA served as vehicle controls. Pain-related behaviors were measured at 1-24 h and 1 week after injection. In addition, VEGFA (10 ng/knee; 5 μL) was intraarticularly injected into the left knee joint cavity of naive mice. The identical amounts of sterile PBS containing 2% BSA served as vehicle control. After IA injection of VEGFA/vehicle for overnight, animals were euthanized, and the knee synovium tissues were isolated for real time-polymerase chain reaction (RT-PCR).

Pazopanib or vandetanib treatments (1.5 mg/mL, 5 μL; twice per week, LC laboratories, Woburn, MA) were done by injection of drugs into the left knee joint cavity of PMM or sham surgery mice at day 5 (treatment for the inflammatory pain stage), week 4 (treatment for early OA) or week 8 (treatment for advanced OA) after surgery. 5% DMSO in PBS (vehicle) in 5 µL volume served as controls.

### Longitudinal behavioral pain measurements

Longitudinal behavioral pain measurements, including mechanical allodynia (Von Frey filament test) and thermal pain assay (Hot plate test) were performed at baseline (before OA induction) and thereafter weekly for 12 weeks.

#### Mechanical allodynia (Von Frey test)

Animals were placed on a perforated metal 'grid' floor (with 5 mm diameter holes placed 7 mm apart) within small Plexiglass cubicles (9x5x5 cm high). A set of eight calibrated Von Frey fibers (Stoelting touch Test Sensory Evaluator Kit) was applied to the plantar surface of the hind paw until the fiber began to bow and then held for 2-3 s. The threshold force required to elicit the withdrawal of the paw was determined. A brisk lifting of the foot was recorded as a positive response. If no response was observed, the filament with the next highest force was applied, while the filament with the next lowest force was applied after a positive response 21,22. Behavioral analyses were performed in a blinded manner.

#### Thermal pain assay (Hot plate test)

The temperature sensitivity of the hind paw was measured using a 'Hotplate Analgesia Meter' (Columbus Instruments, Columbus, USA). Before the experiment, the top plate was cleaned with alcohol, the hot plate was set to 55°C, and the plate was incubated for at least 30 min to allow it to stabilize at 55°C. Then, mice were placed into the hot plate enclosure. Timing was terminated when any of the following behavioral events occurred: licking the hind paw (even once), shaking the hind paw in the air, and jumping. A cutoff latency of 20 s was used to prevent tissue damage 10. Behavioral analyses were performed in a blinded manner.

### RT-PCR

Total RNA was isolated from C28/I2 human immortalized chondrocyte cells or murine knee synovium tissues using the TRIzol method (Invitrogen Corp.) and complementary DNA was synthesized with 1 μg total RNA. Triplicate *RT-PCR* analyses were conducted using a Bio‐Rad CFX Connect system (Bio‐Rad Laboratories, CA) and the SYBR Green method (Bio‐Rad Laboratories). We obtained a threshold cycle (Ct) value and determined relative messenger RNA (mRNA) expression by the ΔΔCt method using the expression of human GAPDH mRNA or mouse β-action mRNA as the internal control. Three independent sets of samples were used to derive the standard deviation of gene expression. Supplementary Table 1 and 2 present the primer sequences.

### Histopathology and Immunofluorescence

Histopathological and immunohistochemical analyses were performed as we previously described 23. Gross knee joint pathology was examined using standard procedures described previously 24. At 12 weeks post-PMM or post-sham surgery, animals were terminally anesthetized and pre-perfused with 4% paraformaldehyde. Entire knee joints were serially sectioned (5 μm) in the sagittal plan and then used for histological and immunofluorescence analyses. Two sections within every consecutive six sections in the entire section set for each knee were stained with safranin-O and fast green and scored by two blinded observers. Briefly, mouse knee joint sections were deparaffinized, hydrated and washed gently in distilled water. These sections were then stained with 0.1% fast green (Sigma-Aldrich) for 1 minute, 1% acetic acid (Sigma-Aldrich) for 30 seconds and 0.1% safranin-O (Sigma-Aldrich) for 30 minutes sequentially. Next, these sections were rinsed in 95% ethanol briefly, dehydrated with ethanol, cleared with xylene and covered with coverslips.

Osteoarthritis Research Society International (OARSI) scoring was used to determine OA grading. For each sample, we analyzed three-levels of each section (50 μM apart) through the medial compartment of the knee. The severity of OA-like phenotype was analyzed using the OARSI scoring system using three-level sections of the joints, including the femoral condyle and tibial plateau, by two blinded observers 25-28. Each knee received a single score representing the maximal score of all its sections. In addition, the lumbar (L3-5) spinal cord and DRG were isolated and postfixed with 4% paraformaldehyde for 48 h and then processed for paraffin embedding and sectioning for immunofluorescence analyses.

Sections were incubated overnight in primary antibodies against pVEGFR1 (1:100, Invitrogen, PA5-99362), pVEGFR2 (1:100, Invitrogen, PA5-105765), pNF-κB (1:100, Invitrogen, 436700), VEGFA (1:500, Abcam, ab185238), NGF (1:500, Abcam, ab52918), ionized calcium-binding adaptor molecule-1 (IBA1, 1:100, Abcam, ab178846), NeuN (1:500, Abcam, ab104224), transient receptor potential vanilloid-1 (TRPV1, 1:200, Abcam, ab6166), RUNX2 (1:100, Abcam, ab192256), PGP9.5 (1:200, Abcam, ab104404), TNFα (1:500, Abcam, ab1793), IL1-β (1:500, Abcam, ab283818), TrkA (1:100, Invitrogen, MA-32123), CGRP (1:500, Abcam, ab81887), BDNF (1:500, Invitrogen, PA1-18357), TrkB (1:500, Sigma-Aldrich, SAB4502034), CCR2 (1:100, Novus Biologicals, NBP235334), MMP13 (1:500, Sigma-Aldrich, MAB13424), and glial fibrillary acidic protein (GFAP, 1:200, Sigma-Aldrich, HPA056030). After washing with PBS 3 times, sections were incubated in Alexa Fluor 546 goat anti-rabbit IgG (1:500, Invitrogen, A-11035) or goat anti-mouse Alexa Fluor 488 IgG (1:500, Invitrogen, A-11029) for 2 h. After washing with PBS 3 times, sections were mounted by mounting medium with DAPI (*Southern Biotech*) and covered with coverslips. Fluorescent images were obtained with a Nikon Eclipse NiE upright microscope (Nikon Instruments Inc., Melville, NY) and associated software.

### Toxicological evaluation of chronic use of IA drug administration

IA injection (twice per week) of pazopanib or vandetanib or vehicle (5% DMSO in PBS, Veh) was done on day 5 (Inflammatory pain stage) after PMM. Body weights of all the animals were measured every week, and behavioral changes were observed. After drug/vehicle treatments for 12 weeks, animals were euthanized, and vital organs such as heart, kidney, liver, and pancreas were isolated. Histopathological studies were performed on these vital organs using hematoxylin-eosin (H&E) staining. Briefly, the specimens were fixed in 10% formalin and processed. After the tissue was embedded in paraffin and sectioned at the 5-µm thickness, sections were dyed with H&E according to the procedure. Photomicrographs were taken with a Nikon light microscope at 200X magnification, and the semi-quantitative toxicological evaluation was performed according to the comparison of the structural changes of the H&E results, as described by Ben et al. 29,30. Each organ tissue resulted in 10 images. For a semi-quantitative comparison of the structural changes, the abnormalities in the tissue sections were graded from 0 (normal structure) to 3 (severe pathological changes).

### Statistics

Data are presented as the mean ± standard error of the mean (SEM). An unpaired Student's *t* test was used to determine P values for comparisons between 2 groups, while one-way analysis of variance (ANOVA) with Tukey-Kramer tests was used for comparisons among more than 2 groups. For cell culture experiments, observations were repeated independently at least three times, and only data from representative experiments are presented. The level of significance was set at P<0.05. Statistical analyses were performed using GraphPad Prism 8 software (GraphPad software, San Diego CA, USA).

## Results

### Roles for VEGF/VEGFR signaling in cartilage degeneration and pain transmission

**Figure 1:**
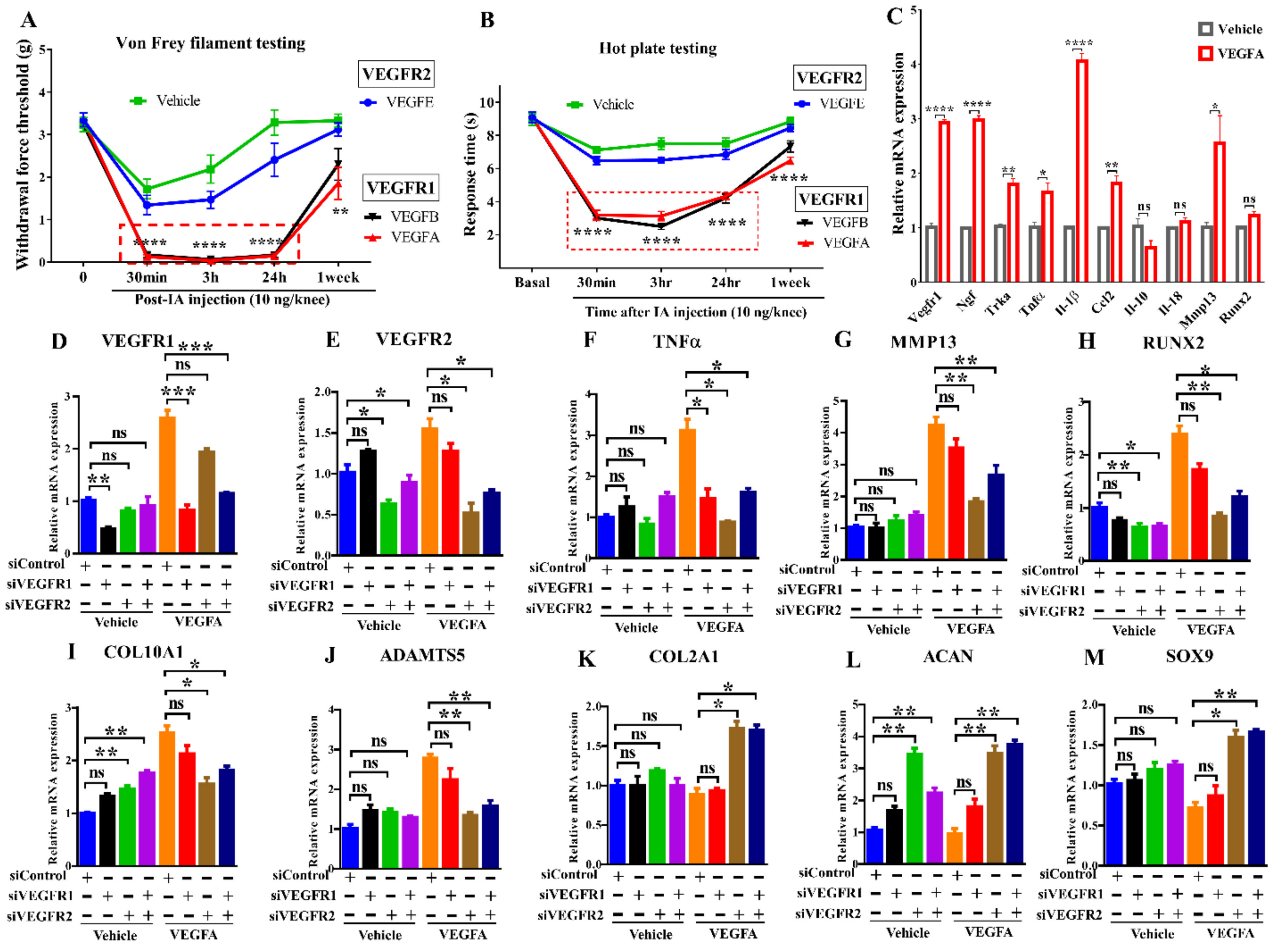
** Distinct roles of VEGFR1 and VEGFR2 signaling in pain transmission and cartilage degeneration** Effects of IA injection of selective ligands for VEGFR1 or VEGFR2 on mechanical allodynia (**A**) and thermal pain assay (**B**) in mice (n = 8/group). The synovium samples were harvested after IA injection of VEGFA for overnight (10 ng/knee, n = 4/group). Real-time PCR analysis was carried out for *Vegfr1, Ngf, Trka, Tnfα, Il-1β, Ccl2, Il-10, Il-18, Mmp13* and *Runx2. β-actin* served as an internal control. The mRNA levels in synovium with the stimulation of VEGFA were normalized to those with vehicle (sterile PBS containing 2% BSA) (**C**). C28/I2 cells were transfected with scrambled siRNA (siControl) or siVEGFR1 or siVEGFR2 in the presence or absence of VEGFA stimulation. Real-time PCR analysis was carried out for *VEGFR1, VEGFR2, TNFα, MMP13, RUNX2, COL10A1, ADAMTS5, COL2A1, ACAN,* and* SOX9,* n = 3 per group **(D-M)**. *GAPDH* served as an internal control. The mRNA levels in cells with VEGFR1 knockdown or VEGFR2 knockdown were normalized to those in control (scrambled siRNA-transfected) cells. Statistical analysis was performed using one-way analysis of variance (ANOVA) with Tukey-Kramer test for (**A, B, D-M**) and unpaired *t* test for (**C**). Data are presented as means ± SEM (*P <0.05, **P < 0.01, ***P < 0.001, ****p<0.0001).

To determine the role of VEGFRs in pain, VEGF ligands selective for either VEGFR1 or VEGFR2 were intraarticularly injected into mouse knees. Mice showed allodynia within 30 minutes upon exposure to VEGF ligands selective for VEGFR1 but not VEGFR2. This result suggests a critical role for VEGFR1 in transmitting pain (Fig. 1A, B). Synovial tissues were harvested after IA injection of VEGFA overnight, VEGFA significantly increases pain markers, proinflammatory cytokines, angiogenic factors, and cartilage degrading enzymes in synovium, including *Vegfr1, Ngf,* and its cognate receptor* Trka,* the cytokines *Il-1β/IL1B* and* Tnfα/TNF, Ccl2*, and *Mmp13*. (Fig. 1C). We cultured C28/I2 human chondrocytes in the presence of VEGFA stimulation. In our quantitative PCR analyses, VEGFA also upregulated catabolic and hypertrophic markers (*MMP13, RUNX2, COL10A1, ADAMTS5*). Silencing *VEGFR2* using siRNAs increased chondrocyte-related anabolic gene expression (*ACAN, COL2A1, SOX9*), and reduced hypertrophic or catabolic genes (*COL10A1, MMP13, RUNX2, ADAMTS5*). Silencing *VEGFR1* did not affect either of these sets of genes. Knockdown of *VEGFR1* or *VEGFR2* inhibited *TNFα* (Fig. 1D-M). These results suggest that VEGFR2 signaling is a principal pathway involved in cartilage degeneration and that VEGFR1 is primarily responsible for VEGF-related pain transmission.

### IA injection of pazopanib shows rapid joint pain relief

We compared the effects of pazopanib, which inhibits VEGFR1 and VEGFR2, and vandetanib, a selective inhibitor of VEGFR2, on pain and pathological progression of OA. We first optimized the concentration and frequency of the drug in our experimental OA model. Von Frey testing showed that pazopanib, but not vandetanib, rapidly reduces pain response (within 30 min of drug injection) and the analgesic effect lasts for three days (Fig. S1A, B). IA injection of 1.5 mg/mL pazopanib showed the most pronounced analgesic effects and higher concentrations did not further decrease pain. Based on these initial data, we surgically induced OA in 12-week-old C57BL/6 mice and commenced IA drug treatment (twice per week) targeting different stages of OA disease progression (Gp:1~Gp:3, Fig. 2A).

**Figure 2:**
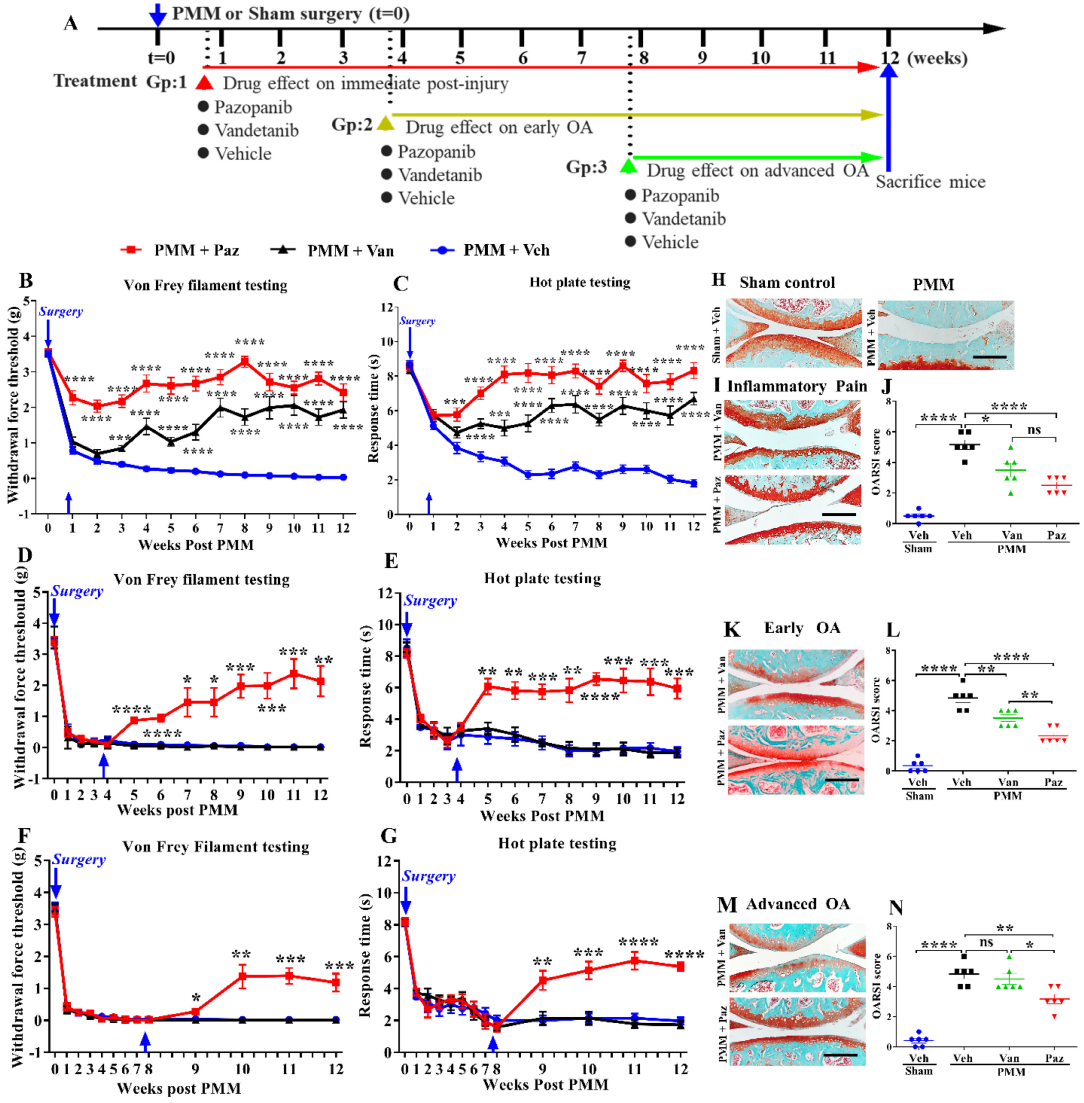
**The effect of pazopanib and vandetanib on OA pain and joint pathology.** Schematic diagram of the research plan (**A**). Pazopanib (Paz, n=18, female n=8, male n=10), vandetanib (Van, n=18, female n=9, male n=9) (both 7.5 µg/knee) or vehicle (Veh, 5% DMSO in PBS, n=17, female n=9, male n=8) were injected intraarticularly (twice per week in 5 µL). Drug treatments began at week 1 (Gp1, inflammatory pain stage), week 4 (Gp2, early OA stage) or week 8 (Gp3, advanced OA stage) after partial medial meniscectomy (PMM). Development of mechanical allodynia (Von Frey filament testing) and thermal pain assay (hot plate testing) in the ipsilateral hind paw when drugs were administered in the inflammatory pain stage (**B, C**), the early OA stage (**D, E**), and the advanced OA stage (**F, G**). Representative images of safranin-O fast green staining of the knee joints for each treatment in different stages of OA after IA injection of pazopanib or vandetanib (**H, I, K, M**). Graphs of average OARSI scores in the different stages of OA (n=6) (**J, L, N**). Statistical analysis was performed using one-way ANOVA with Tukey-Kramer test. Data are presented as means ± SEM (comparisons between groups with or without pazopanib or vandetanib treatment in mice that underwent PMM *p<0.05, **p<0.01, ***p<0.001, ****p<0.0001). Scale bars: 200 μm.

For drug treatment commencing at the inflammatory pain stage (Gp:1), pazopanib immediately reduced pain (p<0.0001). Pain reduction by vandetanib was delayed compared to pazopanib, and required at least three weeks (Fig. 2B, C). Similarly, pazopanib, but not vandetanib, rapidly relieved OA pain when administered later during disease progression (in early (Gp:2) and advanced stages of OA (Gp:3)) (Fig. 2D-G). We compared female and male mice for differences in inflammatory pain stage but did not observe significant sex differences in drug efficacy (Fig. S2A-D).

Next, we examined the therapeutic efficacy of pazopanib and vandetanib on cartilage protection by histopathological analyses of knee joints at 12 weeks. Pathological grading was quantified using the OARSI scoring system. When drug treatments were started within a week post PMM (Gp:1, Fig. 2I), OARSI scores were significantly reduced (Fig. 2J). Pazopanib also protected joints from cartilage degeneration when the treatment started at the early- (Gp:2) or advanced OA stage (Gp:3). However, vandetanib failed to prevent articular cartilage degeneration when the drug was given in the advanced OA stage (Gp:3, Fig. 2K-N). There were no sex differences in OARSI scores between male and female mice (Fig. S2E). Our results indicate that pazopanib exerts dual effects: reducing pain and protecting cartilage from degeneration at all three treatment timepoints (Gp:1~Gp:3). In contrast, vandetanib reduced pain only when started at the inflammatory pain stage (Gp:1). This finding suggests that simultaneously targeting VEGFR1 and VEGFR2 may effectively halt both pain and pathology in OA joints, thus potentially providing an optimal therapeutic strategy.

### Pazopanib inhibits the expression of cartilage catabolic and inflammatory factors

**Figure 3:**
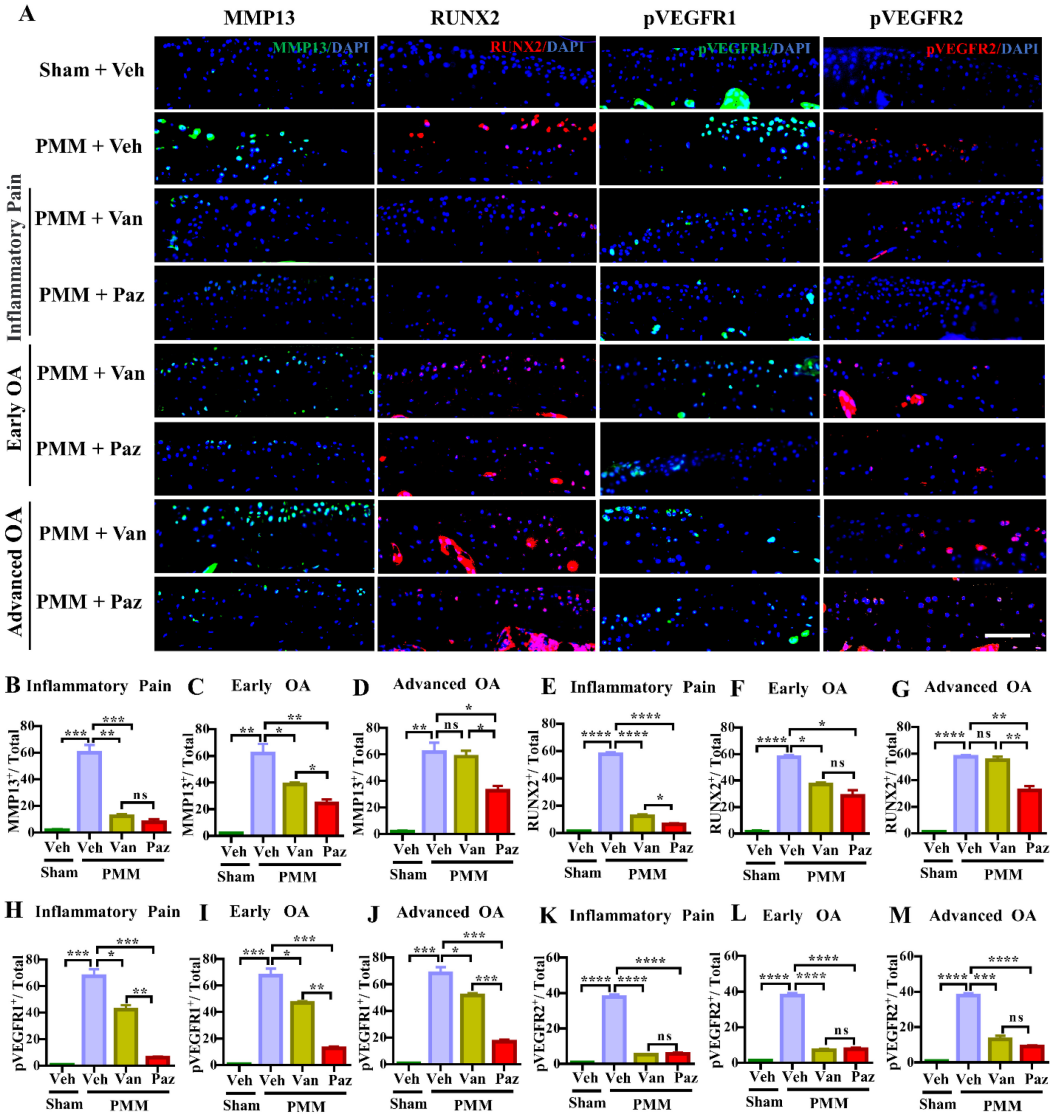
** Inhibition of the activation of VEGFR2 decreases the expression of MMP13 and RUNX2 during OA development and progression.** Immunofluorescence (IF) assays were done in histological sections of cartilage tissues in mice at 12 weeks after partial medial meniscectomy (PMM). IA injection of pazopanib (Paz) or vandetanib (Van) or vehicle (Veh, 5% DMSO in PBS) was performed at week 1 (Gp1, inflammatory pain stage), week 4 (Gp2, early OA stage) or week 8 (Gp3, advanced OA stage) after PMM, twice per week for 12 weeks. Levels of MMP13 (green), RUNX2 (red), pVEGFR1 (green) and pVEGFR2 (red) were examined in knee cartilage by IF microscopy (**A**). Quantitative analysis results for MMP13, RUNX2, pVEGFR1 and pVEGFR2 expression (n=3) (**B-M**). Statistical analysis was conducted using one-way ANOVA followed by the Tukey-Kramer test. *p<0.05, **p<0.01, ***p<0.001, ****p<0.0001 making comparisons between groups with or without pazopanib or vandetanib treatment in mice with PMM. 4*′,*6-diamidino-2-phenylindole (DAPI) stains nuclei blue. Scale bars: 100 μm.

The levels of VEGFR1 and VEGFR2 are increased in chondrocytes from OA patients 31. However, the exact roles of VEGFRs in OA remain uncertain. We examined the activation of VEGFR1 and VEGFR2 as reflected by phosphorylated epitopes in these receptors (pVEGFR1, pVEGFR2) in mouse knee joints at 12 weeks post-PMM. Immunofluorescence microscopy indicated that the activation of VEGFR2 was suppressed, to a similar degree, by either pazopanib or vandetanib treatment. In contrast, pazopanib extensively decreased the activation of VEGFR1 for all three treatment groups (Fig. 3A, 3H-M). Pazopanib reduced MMP13, a potent cartilage degrading enzyme, and RUNX2, a marker for chondrocyte hypertrophy in cartilage (Fig. 3A-G); reduced IL-1β/IL1B and TNFα/TNF in cartilage and synovium (Fig. S3A-M). Vandetanib also suppressed these catabolic markers when treatment started at inflammatory (Gp:1) or early OA stage (Gp:2). These results suggest that the activation of VEGFR2 is biologically linked to the expression of cartilage catabolic factors and plays a role in cartilage degeneration during OA progression.

### Pazopanib suppresses sensory nerve fiber distribution in knee synovium

**Figure 4:**
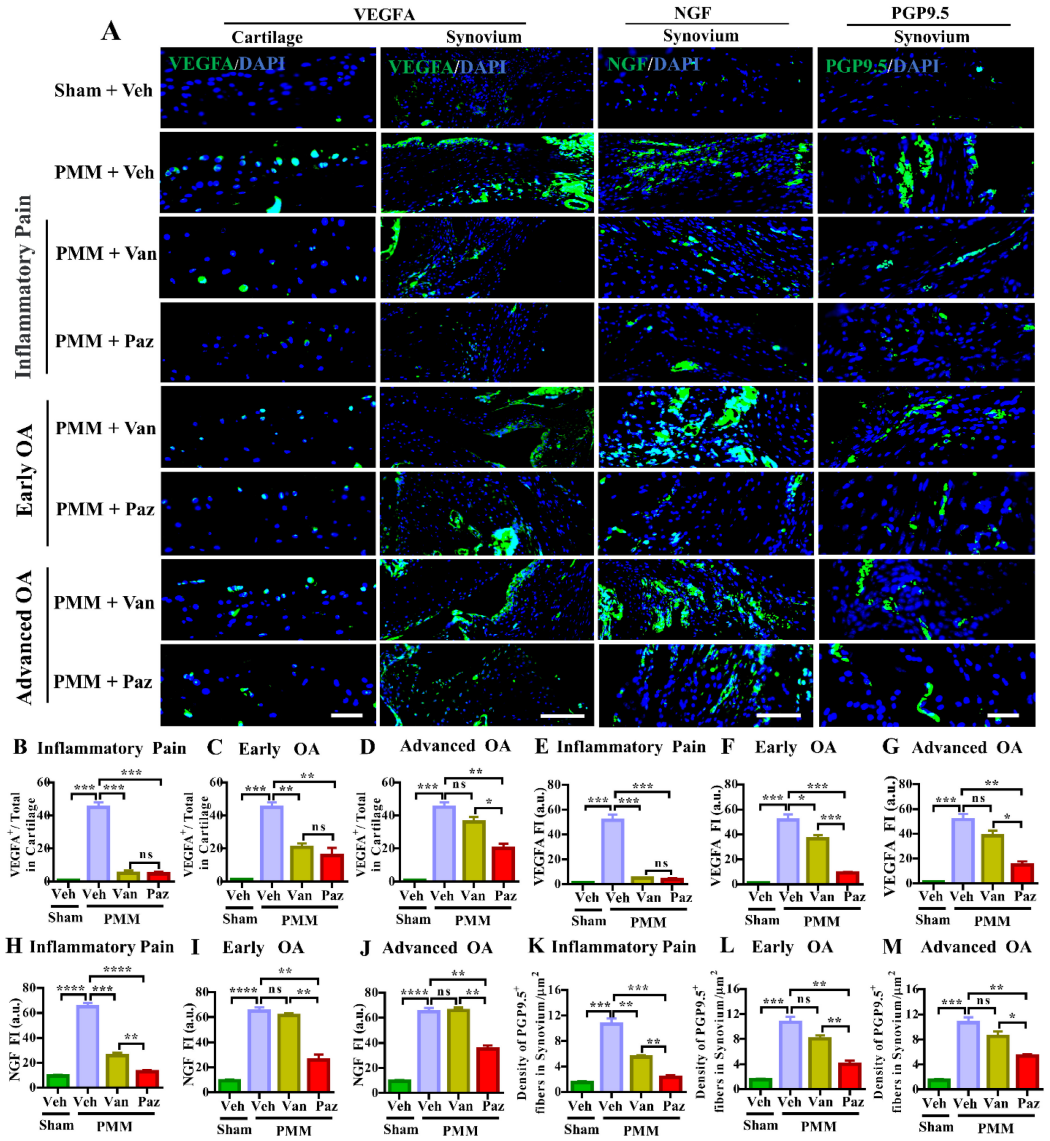
** Pazopanib, not vandetanib, suppressed the number of peripheral nerve fibers sprouting in the synovium-the role of VEGFR1 in sensory nerve distribution in synovium.** Immunofluorescence (IF) assays were performed in histological sections of cartilage and synovial tissues in mice at 12 weeks post-partial medial meniscectomy (PMM). IA administration of drugs (Paz, Van) or vehicle (Veh, 5% DMSO in PBS) commenced at week 1 (Gp1, inflammatory pain stage), week 4 (Gp2, early OA stage) or week 8 (Gp3, advanced OA stage) after PMM twice per week for 12 weeks; and the effect of the drugs or vehicle on the expression of VEGFA (green), NGF (green) and PGP9.5 (green) in cartilage and/or synovium was examined by IF microscopy (**A**). Quantitative analysis showed that VEGFA, NGF and PGP9.5 expressions were significantly increased after PMM (n=3) (**B-M**). Statistical analysis was conducted using one-way ANOVA followed by the Tukey-Kramer test. *p<0.05, **p<0.01, ***p<0.001, ****p<0.0001 are for comparisons between groups with or without drug treatments in mice with PMM*.* 4*′,*6-diamidino-2-phenylindole (DAPI) stains nuclei blue. Scale bars: 100 μm. FI, fluorescence intensity; a.u., arbitrary unit.

We showed that IA injection of VEGFA markedly stimulates NGF and its cognate receptor TrkA in knee joints and DRG sensory neurons 9. Increased NGF/TrkA signaling in synovial tissues and axonal outgrowth that leads to sensory neurite distribution in knee joints appears to be a critical determinant for switching from asymptomatic to painful OA, independent of joint pathology in both patients and a mouse OA model 32. Chemokine (C-C motif) ligand 2/monocyte chemoattractant protein-1 (CCL2/MCP-1) is a potent chemokine for monocytes and other immune cells. Its receptor is C-C chemokine receptor type 2 (CCR2), which is expressed at high levels in monocytes. CCR2 is a seven-transmembrane domain G-protein coupled receptor (GPCR) that acts as the receptor for CCL2, CCL7, and CCL12 33,34. CCL2-CCR2 signaling is potently chemotactic for monocytes and immune cells. CCL2-CCR2 signaling is an essential mediator of pain during the development of experimental OA 35. Ccr2 null mice were protected from persistent pain behaviors in the surgical-induced OA model 36. Therefore, we assessed nerve fiber density and the expression levels of VEGFA, NGF, TrkA and CCR2 in the knee synovium with or without drug treatments by immunofluorescence microscopy. Increased VEGFA level was observed in knee cartilage and synovium in the vehicle-treated mice subjected to PMM (Fig. 4A). Pazopanib and vandetanib treatments targeting the inflammatory (Gp:1), and the early OA (Gp:2) decreased VEGFA in cartilage. However, only pazopanib, not vandetanib, reduced NGF, TrkA, CCR2 and the distribution of sensory nerve fibers (detected by PGP9.5) in synovium for all three treatment groups (Gp:1~Gp:3) (Fig. 4, Fig. S4). These data corroborate our pain tests (Fig. 2) in which pazopanib reduces pain at all stages of OA treatments. Collectively, our results suggest that pazopanib reduces pain by suppressing key pain mediators (NGF/TrkA, CCR2 and VEGFA) 22,37, leading to reduction in levels of expression of sensory nerve fibers in knee synovium.

### Pazopanib reduces pain-related markers expression in DRG sensory neurons

**Figure 5:**
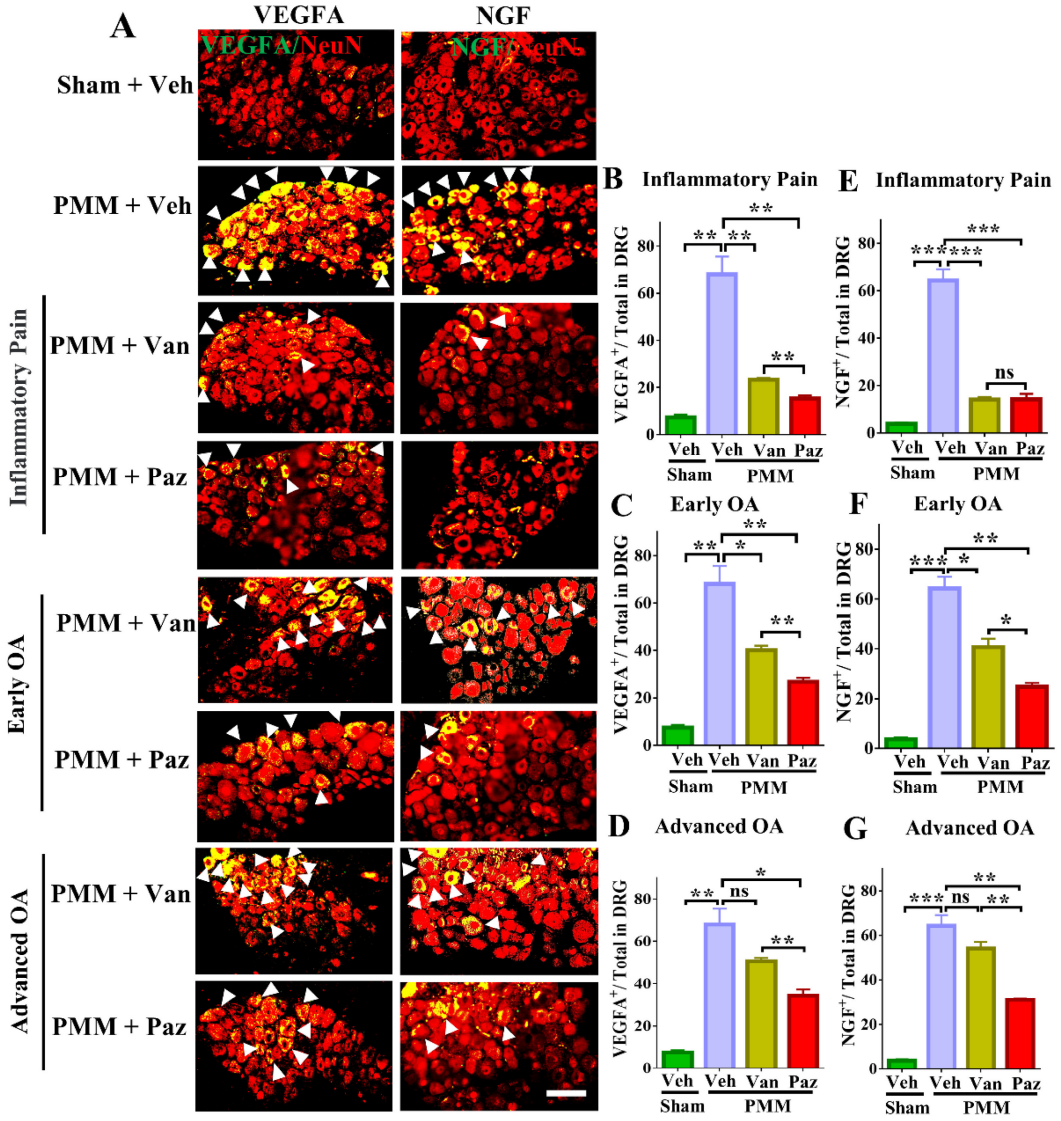
** Pazopanib reduced the expression of NGF and VEGFA in sensory neurons (DRG), which were associated with OA pain.** Innervating lumbar DRG (L3-L5) were assessed for expression of VEGFA and NGF in sensory neurons (using NeuN as a neuron marker, red) by dual immunofluorescence (IF), arrows indicate DRG neurons positive for VEGFA (green) or NGF (green) (**A**). IF assays were performed in histological sections of DRG tissues in mice at 12 weeks post-partial medial meniscectomy (PMM). IA injection of pazopanib (Paz), vandetanib (Van) or vehicle (Veh, 5% DMSO in PBS) was done at week 1 (Gp1, inflammatory pain stage), week 4 (Gp2, early OA stage) or week 8 (Gp3, advanced OA stage) after PMM, twice per week for 12 weeks, and the effect of drugs or vehicle on the expression of VEGFA and NGF in the DRG was determined. Quantitative analysis showed that VEGFA and NGF expression was significantly increased after PMM (n=3) (**B-G**). Statistical analysis was conducted using one-way ANOVA followed by the Tukey-Kramer test. *p<0.05, **p<0.01, ***p<0.001 for comparisons between groups with or without drug treatments in mice with PMM. Scale bars: 100 μm.

We tested the effects of drugs on pain-related markers expression (NGF, TrkA VEGFA, CGRP, CCR2 and BDNF/TrkB) in DRG sensory neurons by dual staining with NeuN, a neuron-specific nuclear protein (gene symbol RBFOX3). Expression of NGF, TrkA, VEGFA, CGRP, CCR2 and BDNF/TrkB was increased in DRG sensory neurons of mice with OA pain. Pazopanib counteracted these changes, significantly decreasing NGF, TrkA, VEGFA, CGRP, CCR2 and BDNF/TrkB protein expression in all three treatment groups (Gp:1~Gp:3). Although NGF, TrkA, VEGFA, CGRP, CCR2 and BDNF/TrkB levels were decreased when vandetanib treatment started at the inflammatory or early OA stage, it failed to reduce these pain mediators when the treatments commenced at the advanced OA stage (Fig. 5, Fig. S5). Thus, the expression of VEGFA and NGF/TrkA, CGRP, CCR2 and BDNF/TrkB is not directly related to vandetanib treatment.

### Pazopanib inhibits the activation of VEGFR1 in DRG sensory neurons

**Figure 6:**
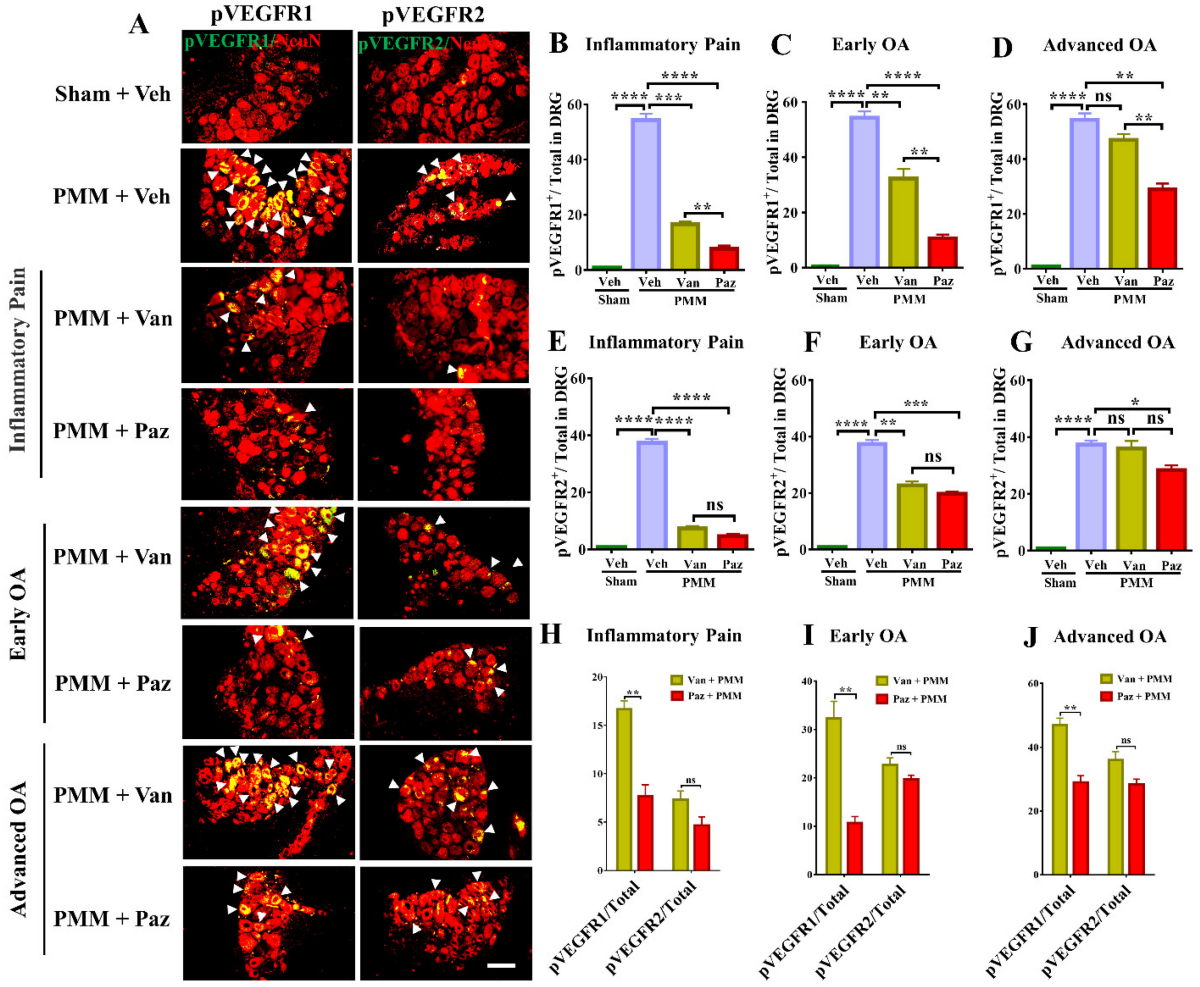
** Pazopanib reduced the activation of VEGFR1 in DRG sensory neurons.** Innervating lumbar DRG (L3/L5) were assessed for activation of VEGFR1 and VEGFR2 in DRG sensory neurons by dual immunostaining with NeuN (red), a neuron marker, arrows indicate DRG neurons positive for VEGFR1 (green) and VEGFR2 (green) (**A**). IF assays were performed in histological sections of DRG tissues in mice 12 weeks post-partial medial meniscectomy (PMM). IA injection of pazopanib (Paz), vandetanib (Van) or vehicle (Veh, 5% DMSO in PBS) was done at week 1 (Gp1, inflammatory pain stage), week 4 (Gp2, early OA stage) or week 8 (Gp3, advanced OA stage) after PMM, twice per week for 12 weeks. Quantitative analysis of IF microscopy demonstrated that activation of VEGFR1 (pVEGFR1) and VEGFR2 (pVEGFR2) was significantly increased after PMM (n=3) (**B-G**). Activation of VEGFR1 was markedly suppressed by pazopanib treatment while pazopanib and vandetanib inhibited the activation of VEGFR2 with similar magnitudes (**H-J**). Statistical analysis was conducted using one-way ANOVA followed by the Tukey-Kramer test. *p<0.05, **p<0.01, ***p<0.001, ****p<0.0001 and we compared groups with or without pazopanib or vandetanib treatment in mice that underwent PMM. Scale bars: 100 μm.

To determine the molecular mechanism by which IA injection of pazopanib reduces OA-associated joint pain, we examined the correlation of VEGFR activity and the *in-situ* presence of pain-responsive molecules in DRG sensory neurons with or without drugs. In our dual immunostaining experiments with the neuron marker NeuN, VEGFR1 activation by phosphorylation (pVEGFR1) was markedly increased in innervating DRG sensory neurons in mice with chronic OA pain. Levels of pVEGFR2 also increased but the elevation of pVEGFR1 was considerably higher in magnitude (Fig. 6A, H-J). Pazopanib treatments reduced pVEGFR1 levels significantly for all three treatment groups. However, vandetanib predominantly suppressed VEGFR2 levels except when drug treatment started at the inflammatory pain and early OA stages (Fig. 6). These findings suggest that VEGFR1 may modulate sensory neuronal plasticity to modulate OA pain sensitization.

Next, we examined TRPV1 levels, a downstream pain-related marker of VEGFR1^38^. TRPV1 was upregulated in DRG sensory neurons of animals with chronic joint pain; This upregulation was reversed by pazopanib for all three treatment time points (Gp:1~Gp:3). In contrast, vandetanib suppressed TRPV1 expression only when the treatment started at the inflammatory OA stage (Fig. S6). This finding is important because the blockade of VEGFR1 expression in DRG sensory neurons correlates with the suppression of TRPV1, which is associated with thermal nociception.

### IA administration of pazopanib suppresses the NF-κB, microglial and astroglial activation in the spinal cord of the experimental OA animals

**Figure 7:**
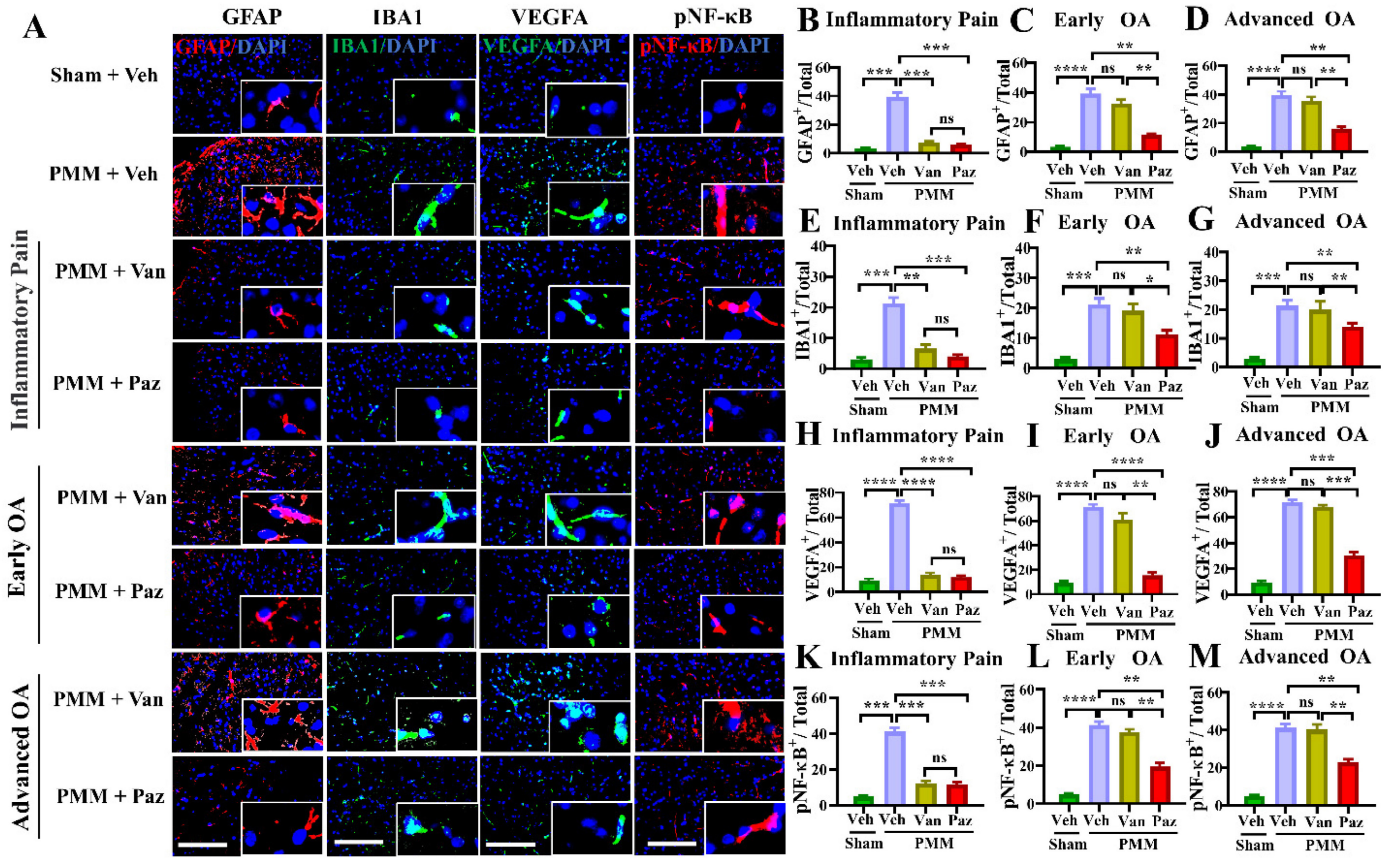
** Pazopanib reduces spinal NF-kB-glial axis activation.** Spinal cords (L3-L5) were assessed for the expression of pNF-κB and VEGFA and the reactivity of astrocytes and microglia using double immunofluorescence (IF). IF assays were performed in histological sections of spinal cord tissues in mice at 12 weeks post-partial medial meniscectomy (PMM). IA injection of pazopanib (Paz), vandetanib (Van) or vehicle (Veh, 5% DMSO in PBS) was performed at week 1 (Gp1, inflammatory pain stage), week 4 (Gp2, early OA stage) or week 8 (Gp3, advanced OA stage) after PMM, twice per week for 12 weeks, and the effect of pazopanib, vandetanib or vehicle on the expression of pNF-κB and VEGFA and the reactivity of astrocytes and microglia in the lumbar spinal dorsal horns were observed; the expression of GFAP (an astrocyte marker, red), IBA1 (a microglia marker, green), pNF-κB (red) and VEGFA (green) was examined by IF microscopy (**A**). Quantitative analysis demonstrated that the expression of GFAP, IBA1, VEGFA and NF-κB activation were significantly increased after PMM (n=3) (**B-M**). Statistical analysis was conducted using one-way ANOVA followed by the Tukey-Kramer test. *p<0.05, **p<0.01, ***p<0.001, ****p<0.0001 comparisons between groups with or without pazopanib or vandetanib treatment in mice that underwent PMM. 4*′,*6-diamidino-2-phenylindole (DAPI) stains nuclei blue. Scale bars: 100 μm.

Spinal glial cells are known to play a role in sensitization to neuropathic pain 39-42. NF-κB (NFKB1) is a crucial regulator of glial activation 40,43,44. We determined whether knee OA pain correlates with spinal glial activation, and whether drug treatments can modulate the NF-κB-glial axis. Immunofluorescence microscopy studies showed increased levels of NF-κB activation (pNF-κB), VEGFA, GFAP (a marker for astroglial activation), and of IBA1 (a marker for microglial activation). These were increased in the ipsilateral spinal dorsal horn of mice with OA compared to those in sham mice (Fig. 7).

**Figure 8:**
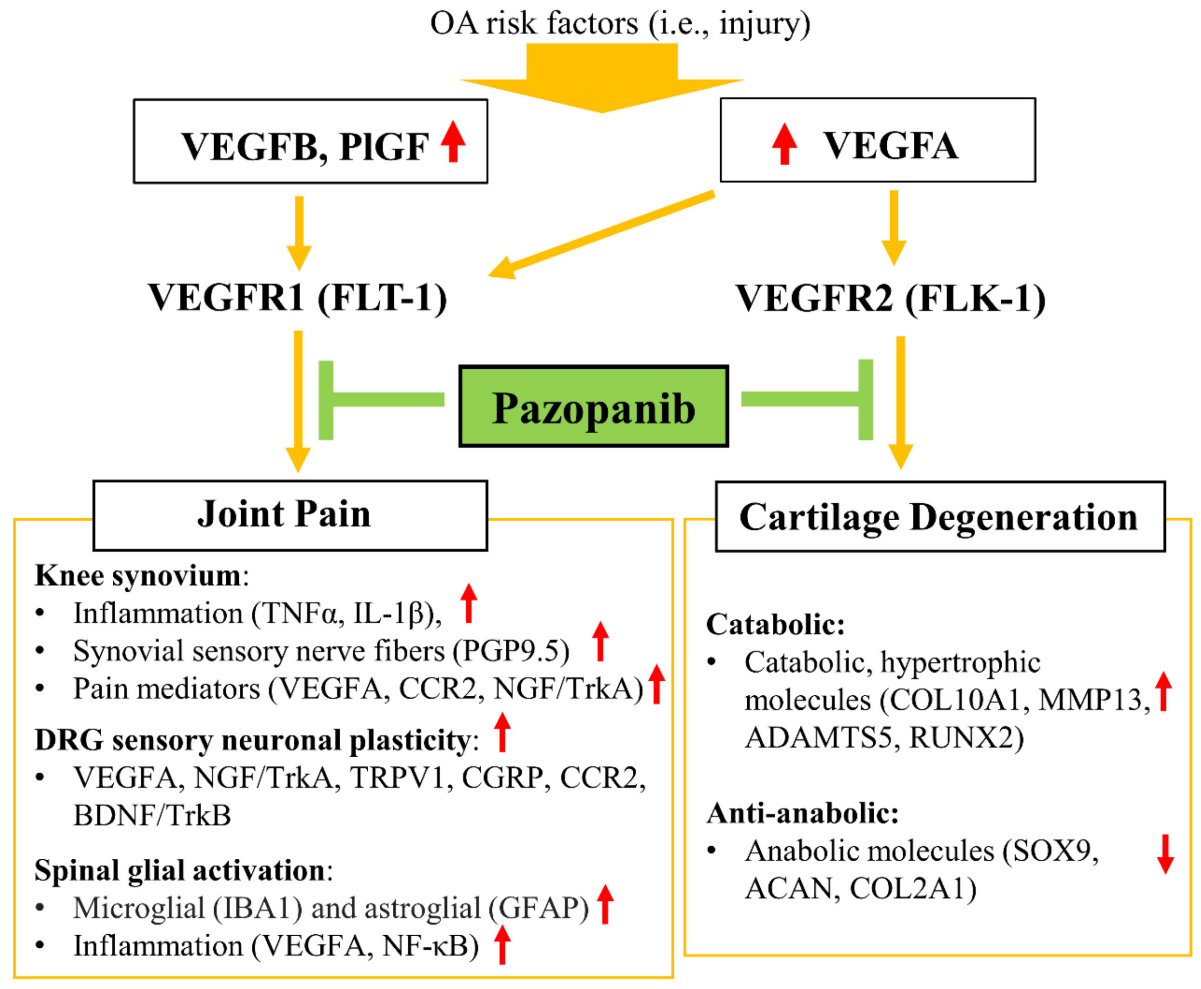
Schematic diagram of the role of pazopanib in pain relief and joint protection from cartilage degeneration by simultaneous inhibition of VEGFR1 and VEGFR2.

For mice treated with pazopanib, the levels of pNF-κB, VEGFA, and the number of activated glial cells were reduced significantly for all three treatment groups (Fig. 7). Also, IA injection of pazopanib markedly attenuated pVEGFR1 levels in the spinal dorsal horn (Fig. S7). In contrast, vandetanib failed to reduce the levels of these markers except when the treatment started at the inflammatory pain stage (Gp:1). These results suggest that VEGFR1 activation in this context has a role beyond angiogenesis and is involved in nociceptive synaptic transmission in the spinal dorsal horn (central sensitization), which leads to sustained pain states during the development of OA (Fig. 8).

From a translational perspective and in consideration of potential adverse effects upon transitioning into clinical trials in the future, local (IA) injection avoids side effects resulting from immunological and systemic intervention in VEGF signaling. Our toxicological evaluation of long-term use of drug injections showed no signs of toxicity for pazopanib or vandetanib (7.5 μg**/** knee joint, twice per week) (Fig. S8), which is encouraging for possible clinical applications.

## Discussion

Increased VEGF expression in synovial fluids of OA patients has been directly correlated with higher pain scores and OA progression**.** Elevated VEGF levels have also been linked to cartilage degeneration and inflammatory responses 6,38,45. Moreover, VEGF is a potent proangiogenic factor and a key mediator of neovascularization 5,46,47. Although the specific functions of VEGF ligands or VEGFRs had not been sufficiently explored, our study now presents several converging lines of evidence for the therapeutic concept that VEGFRs are viable targets for OA interventions using pharmacological inhibitors. The key insight of our work is that pharmacological inhibitors that target VEGFR1 and VEGFR2 simultaneously in peripheral tissues can attenuate nociceptive sensitization and counteract cartilage degeneration, thus alleviating pain and structural damage in articulating joints. This may not only lead to improved pain and function in patients, but also decrease the utilization of expensive surgical interventions such as joint arthroplasty.

When mouse knee joints were exposed to ligands selective for VEGFR1 but not those selective for VEGFR2, the animals responded sharply (within 30 min) to mechanical and heat stimulation as behavioral pain responses. This finding clearly indicates that locally expressed VEGFR1 is involved in OA pain. The distinct roles of VEGFR1 and VEGFR2 in OA progression are also evident from results obtained for IA administration of pazopanib, an inhibitor of VEGFR1 and VEGFR2, and vandetanib, which selectively inhibits VEGFR2. VEGFR1 was upregulated in knee joints, DRG sensory neurons, and spinal cord dorsal horn in our experimental murine OA pain model. The outcomes from this series of experiments suggest that VEGFA induces nociceptive sensitization via VEGFR1 in peripheral sensory neurons (PSNs). Pazopanib reduced the expression of inflammatory factors (TNFα and IL-1β) and neuron growth-promoting factors (NGF, VEGFA) in knee synovium. Pazopanib also **(i)** decreased expression of VEGFA and NGF in DRG, while inhibiting **(ii)** the subsequent phosphorylation of VEGFR1, **(iii)** activation of NF-κB signaling, as well as **(iv)** the biological activity of spinal astrocytes and microglia in the central nervous system (CNS).

Studies have shown that VEGFR1 modulates TRPV1 activation in nociceptive terminals 13,14,48. Our studies show that pazopanib-mediated amelioration of thermal hyperalgesia is closely corroborated with the markedly reduced activation of TRPV1 in DRG sensory neurons. These findings suggest that IA injection of pazopanib inhibits VEGFR1 at the level of peripheral sensory neurons to attenuate OA-induced neuronal plasticity, consequently blocking joint pain sensation. Consistent with results from our group and others, transgenic mice lacking an RTK signaling domain for VEGFR1 (*Vegfr1-Tk-/-),* VEGFR1 signaling in nociceptive neurons (*SNS-Vegfr1-/-),* or VEGFR1 signaling in sensory neurons (*Adv-Vegfr1-/-*) showed no thermal or mechanical hyperalgesia in response to joint degeneration or VEGFA stimulation 13,49,50. Furthermore, intrathecal injection of a monoclonal antibody against VEGFR1, but not VEGFR2, reduced chronic OA joint pain 10. Thus, VEGFR1 may be the major driver of joint pain transmission in our experimental mouse OA model.

Specific genetic silencing of *VEGFR2* but not *VEGFR1* selectively elevates expression of chondrocyte-related anabolic genes that were suppressed by exogenous VEGFA (e.g., *ACAN*, *COL2A1*, and *SOX9*), and impeded expression of hypertrophic genes (e.g., *COL10A1*, *MMP13*, *RUNX2*, and *ADAMTS5*) that mediate cartilage remodeling. Pazopanib or vandetanib also suppressed the expression of *MMP13* and *RUNX2* in articular cartilage. These results extend our previous studies showing that oral, rather than IA administration of the VEGFR2 kinase inhibitor vandetanib, also attenuates OA progression by reducing pVEGFR2 in articular cartilage and synovial cells 9. Collectively, our findings demonstrate that VEGFR2 has a crucial function in cartilage degeneration in OA.

Remarkably, our results showed a functional dichotomy of the two main lines of VEGF signaling in sensory nerves and cartilage degeneration. VEGFR1 but not VEGFR2 contributes to nerve sensitization and, as such, has a selective role in cancer pain 13. Other studies showed that decreased expression of VEGFR2 in DRG attenuates neuropathic pain 51. Our finding indicates that VEGFR1 is involved in knee OA pain transmission, while VEGFR2 is responsible for cartilage degeneration. Inhibition of VEGFR2 using vandetanib did not mitigate severe pain in mice with early or advanced OA. Interestingly, Vandetanib ameliorated OA pain only when treatment commenced in the inflammatory OA stage and with at least three consecutive weeks of drug administration. This vandetanib-mediated pain relief was associated with inhibition of cartilage degeneration, by reducing catabolic and hypertrophic mRNA markers (*MMP13* and *RUNX2*), and eventually promoted tissue regeneration, rather than directly interfering with pain transmission. These effects can explain why OA pain is refractory to vandetanib while this inhibitor failed to preserve cartilage (e.g., due to the lack of cellularity) when drug treatments were initiated at early or advanced stages of OA. However, pazopanib treatments immediately reduced OA pain at all three stages of OA development. Collectively, our results indicate that VEGFR1 plays a vital role in OA pain transmission, while VEGFR2 triggers cartilage degeneration without a direct contribution to OA pain (Fig. 8).

Pain is the most common reason that patients suffering from OA seek medical treatment for their condition. VEGF/VEGFR1 signaling directly sensitizes nociceptors. Studies have suggested that VEGF/VEGFR1 signaling regulates NGF/TrkA expression 6,14,52,53. In the current study, the blockade of VEGF/VEGFR1 by IA injection of pazopanib downregulated NGF in knee joint synovium and DRG sensory neurons in our experimental OA model. Pazopanib reduced VEGF/VEGFR1 signaling in the spinal dorsal horn, inhibiting NF-κB activity and consequently suppressing spinal astroglial activation as the spinal mechanism of pazopanib-reduced chronic OA pain sensation. IA injection of VEGFA caused acute pain, retrogradely transported to the DRG sensory neurons via VEGFR1 that presents sensory nerve terminals 10. Significantly, our present studies show that IA injection of pazopanib reduces retrograde transport of the VEGF/VEGFR1 complex and reduces synovial sensory nerve terminals, which are directly correlated with OA-induced hyperalgesia both in humans and in our OA animal model.

Importantly, intrathecal injection of a monoclonal antibody (mAb) against VEGFR1, but not VEGFR2, reduced chronic OA joint pain 10. These findings suggest that pazopanib inhibits the activation of NGF signaling in joint synovium, DRG sensory neurons and suppresses spinal glial activity by arresting the VEGF/VEGFR1-NFκB signaling axis, which is correlated with OA joint pain. In addition, other studies showed direct nociceptive effects of VEGFA/VEGFR1 signaling by modulating the trafficking of TRPV1, which subsequently sensitizes transducers of heat, pressure, or chemical stimuli in nociceptive terminals 13,14,54. VEGFR1 activates diverse kinases, such as PLCγ (PLCG1), PI3K, and SRC kinase, that sensitize transducers of heat, pressure, and chemical stimuli in nociceptive terminals, such as those expressing TRPV1 13. We found that pazopanib relieved thermal hyperalgesia in our OA animal model, concomitant with reduced VEGF/VEGFR1-TRPV1 activity in DRG sensory neurons (Fig. 6, Fig. S6).

Furthermore, given that our findings in different sets of experiments showed the redundant and compensatory roles among VEGFR1 ligands in knee joint pain, targeting individual ligands may be less efficacious than targeting the receptors they converge on. The administration of pazopanib, an inhibitor of VEGFR1 and VEGFR2, immediately relieved trauma-induced OA pain and attenuated cartilage degeneration. Our current studies provide strong evidence for the concept that targeting VEGFRs may have major translational potential for OA treatment.

As a prelude to clinical translation, we examined toxicological effects of chronic local administration of pazopanib or vandetanib. These studies are important because patients with systemic administration of pazopanib or vandetanib reported major side effects, including kidney injury 55,56 or hepatotoxicity 57. Local (IA) injection avoids side effects resulting from immunological and systemic intervention in VEGF signaling. Much to our encouragement, toxicological evaluation of chronic IA injections revealed no toxicity for either drug (Fig. S8). We also found no significant sex differences in drug efficacy with long-term use of IA injections of these drugs (Fig. S2).

The current study has several limitations. First, the drugs in our study were administered twice per week, which is not practical but also not insurmountable for clinical settings. It will be necessary to develop drug formulations for pazopanib that provide prolonged drug efficacy to reduce the frequency of IA injections. Second, although pazopanib rapidly reduces joint pain and protects cartilage, this inhibitor may not be ideal as a treatment option for advanced OA stage due to the lack of cellularity in cartilaginous tissues. Third, a robust clinical understanding of the full spectrum of treatment effects of pazopanib on OA will require a deeper understanding of the precise functions of VEGFR1 in DRGs and spinal astrocytes in persistent OA pain.

Taken together, simultaneous inhibition of VEGFR1 and VEGFR2 through local injection of pazopanib therapy is a promising therapeutic strategy that may act as an ideal OADMD by immediately reducing joint pain and protecting joint tissues from degeneration, that leads to the gradual regeneration of cartilage.

## Supplementary Material

Supplementary figures and tables.

## Abbreviations

VEGF: vascular endothelial growth factor
OA: osteoarthritis
VEGFR: VEGF receptor
IA: Intraarticular
OADMD: OA-disease-modifying-drug
PGF/PlGF: placental growth factor
qPCR: quantitative polymerase chain reaction
OARSI: Osteoarthritis Research Society International
PMM: partial medial meniscectomy
IL-1β/IL1B: interleukin-1β
TNFα/TNF: tumor necrosis factor-alpha
DRG: dorsal root ganglia
CGRP: calcitonin gene-related peptide
BDNF: brain-derived neurotrophic factor
CCR2: C-C motif chemokine receptor 2
TrkB: tyrosine kinase receptor B
TRPV1: transient receptor potential vanilloid-1
GFAP: glial fibrillary acidic protein
IBA1: ionized calcium-binding adaptor molecule-1
PSNs: peripheral sensory neurons
CNS: central nervous system
H&E: hematoxylin-eosin

## References

[1] Yu H, Huang T, Lu WW, Tong L, Chen D (2022). Osteoarthritis Pain. *Int J Mol Sci*.

[2] Wei Y (2021). Phospholipase A(2) inhibitor-loaded micellar nanoparticles attenuate inflammation and mitigate osteoarthritis progression. *Sci Adv*.

[3] Chen L, Ni Z, Huang J, Zhang R, Zhang J, Zhang B, Kuang L, Sun X, Zhang D, Su N (2021). Long term usage of dexamethasone accelerating accelerates the initiation of osteoarthritis via enhancing chondrocyte apoptosis and the extracellular matrix calcification and apoptosis of chondrocytes. Int. J. Biol. Sci. *17*, 4140-4153.

[4] Liao L, Zhang S, Zhao L, Chang X, Han L, Huang J, Chen D (2020). Acute Synovitis after Trauma Precedes and is Associated with Osteoarthritis Onset and Progression. Int. J. Biol. Sci. *16*, 970-980.

[5] Pfander D, Körtje D, Zimmermann R (2001). Vascular endothelial growth factor in articular cartilage of healthy and osteoarthritic human knee joints. *Ann Rheum Dis*.

[6] Hamilton JL, Nagao M, Levine BR (2016). Targeting VEGF and Its Receptors for the Treatment of Osteoarthritis and Associated Pain. *J Bone Miner Res*.

[7] Shibuya M (2011). Vascular Endothelial Growth Factor (VEGF) and Its Receptor (VEGFR) Signaling in Angiogenesis: A Crucial Target for Anti- and Pro-Angiogenic Therapies. *Genes Cancer*.

[8] Ludin A, Sela JJ, Schroeder A (2013). Injection of vascular endothelial growth factor into knee joints induces osteoarthritis in mice. *Osteoarthritis Cartilage*.

[9] Nagao M, Hamilton JL, Kc R (2017). Vascular Endothelial Growth Factor in Cartilage Development and Osteoarthritis. *Sci Rep*.

[10] Das V, Kc R, Li X (2018). Blockade of Vascular Endothelial Growth Factor Receptor-1 (Flt-1), Reveals a Novel Analgesic for Osteoarthritis-Induced Joint Pain. *Gene Rep*.

[11] MacDonald IJ, Liu SC, Su CM (2018). Implications of Angiogenesis Involvement in Arthritis. *Int J Mol Sci*.

[12] Koch S, Claesson-Welsh L (2012). Signal transduction by vascular endothelial growth factor receptors. *Cold Spring Harb Perspect Med*.

[13] Selvaraj D, Gangadharan V, Michalski CW (2015). A Functional Role for VEGFR1 Expressed in Peripheral Sensory Neurons in Cancer Pain. *Cancer Cell*.

[14] Llorián-Salvador M, González-Rodríguez S (2018). Painful Understanding of VEGF. *Front Pharmacol*.

[15] Ferrara N (2004). Vascular endothelial growth factor: basic science and clinical progress. *Endocr Rev*.

[16] Enomoto H, Inoki I, Komiya K (2003). Vascular endothelial growth factor isoforms and their receptors are expressed in human osteoarthritic cartilage. *Am J Pathol*.

[17] O-Sullivan I, Natarajan Anbazhagan A, Singh G (2022). Lactobacillus acidophilus Mitigates Osteoarthritis-Associated Pain, Cartilage Disintegration and Gut Microbiota Dysbiosis in an Experimental Murine OA Model. Biomedicines.

[18] Podar K, Tonon G, Sattler M (2006). The small-molecule VEGF receptor inhibitor pazopanib (GW786034B) targets both tumor and endothelial cells in multiple myeloma. *Proc Natl Acad Sci U S A*.

[19] Harris PA (2008). Discovery of 5-[[4-[(2,3-dimethyl-2H-indazol-6-yl)methylamino]-2-pyrimidinyl]amino]-2-methyl-benzenesulfonamide (Pazopanib), a novel and potent vascular endothelial growth factor receptor inhibitor. *J Med Chem*.

[20] Alao JP, Michlikova S, Dinér P (2014). Selective inhibition of RET mediated cell proliferation *in vitro* by the kinase inhibitor SPP86. *BMC Cancer*.

[21] Deuis JR, Dvorakova LS, Vetter I (2017). Methods Used to Evaluate Pain Behaviors in Rodents. *Front Mol Neurosci*.

[22] Kc R, Li X, Kroin JS (2016). PKCδ null mutations in a mouse model of osteoarthritis alter osteoarthritic pain independently of joint pathology by augmenting NGF/TrkA-induced axonal outgrowth. *Ann Rheum Dis*.

[23] Kc R, Li X, Voigt RM (2015). Environmental disruption of circadian rhythm predisposes mice to osteoarthritis-like changes in knee joint. *J Cell Physiol*.

[24] Im HJ, Kim JS, Li X (2010). Alteration of sensory neurons and spinal response to an experimental osteoarthritis pain model. *Arthritis Rheum*.

[25] Wei Y, Luo L, Gui T (2021). Targeting cartilage EGFR pathway for osteoarthritis treatment. *Sci Transl Med*.

[26] Lu J (2017). MicroRNA-218-5p as a Potential Target for the Treatment of Human Osteoarthritis. *Mol Ther*.

[27] Sun AR, Wu X, Liu B (2019). Pro-resolving lipid mediator ameliorates obesity induced osteoarthritis by regulating synovial macrophage polarisation. *Sci Rep*.

[28] Li J, Zhang B, Liu WX (2020). Metformin limits osteoarthritis development and progression through activation of AMPK signalling. *Ann Rheum Dis*.

[29] Ben Ali R, Ben Othman A, Bokri K (2017). Synthesis and evaluation of analgesic, behavioral effects and chronic toxicity of the new 3,5-diaminopyrazole and its precursor the thiocyanoacetamide. *Biomed Pharmacother*.

[30] Ibrahim KE, Al-Mutary MG, Bakhiet AO (2018). Histopathology of the Liver, Kidney, and Spleen of Mice Exposed to Gold Nanoparticles. *Molecules*.

[31] Fay J, Varoga D, Wruck CJ (2006). Reactive oxygen species induce expression of vascular endothelial growth factor in chondrocytes and human articular cartilage explants. *Arthritis Res Ther*.

[32] Hu Q, Ecker M (2021). Overview of MMP-13 as a Promising Target for the Treatment of Osteoarthritis. *Int J Mol Sci*.

[33] Lim SY, Yuzhalin AE, Gordon-Weeks AN (2016). Targeting the CCL2-CCR2 signaling axis in cancer metastasis. Oncotarget.

[34] Miller RE, Malfait AM (2017). Can we target CCR2 to treat osteoarthritis? The trick is in the timing. Osteoarthritis Cartilage.

[35] Ishihara S, Obeidat AM, Wokosin DL (2021). The role of intra-articular neuronal CCR2 receptors in knee joint pain associated with experimental osteoarthritis in mice. Arthritis Res Ther.

[36] Miller RE, Tran PB, Das R (2012). CCR2 chemokine receptor signaling mediates pain in experimental osteoarthritis. Proc Natl Acad Sci U S A.

[37] Colangelo AM, Cirillo G, Alberghina L (2019). Neural plasticity and adult neurogenesis: the deep biology perspective. *Neural Regen Res*.

[38] Di Nicola V (2020). Degenerative osteoarthritis a reversible chronic disease. *Regen Ther*.

[39] Wei Z, Fei Y, Su W (2019). Emerging Role of Schwann Cells in Neuropathic Pain: Receptors, Glial Mediators and Myelination. *Front Cell Neurosci*.

[40] Katz JN, Arant KR, Loeser RF (2021). Diagnosis and Treatment of Hip and Knee Osteoarthritis: A Review. *JAMA*.

[41] Gwak YS, Hulsebosch CE, Leem JW (2017). Neuronal-Glial Interactions Maintain Chronic Neuropathic Pain after Spinal Cord Injury. *Neural Plast*.

[42] Jha MK, Jeon S, Suk K (2012). Glia as a Link between Neuroinflammation and Neuropathic Pain. *Immune Netw*.

[43] Mattson MP, Camandola S (2001). NF-kappaB in neuronal plasticity and neurodegenerative disorders. *J Clin Invest*.

[44] Shih RH, Wang CY, Yang CM (2015). NF-kappaB Signaling Pathways in Neurological Inflammation: A Mini Review. *Front Mol Neurosci*.

[45] Wojdasiewicz P, Poniatowski ŁA, Szukiewicz D (2014). The role of inflammatory and anti-inflammatory cytokines in the pathogenesis of osteoarthritis. *Mediators Inflamm*.

[46] Mettu PS, Allingham MJ, Cousins SW (2021). Incomplete response to Anti-VEGF therapy in neovascular AMD: Exploring disease mechanisms and therapeutic opportunities. *Prog Retin Eye Res*.

[47] Simons M, Gordon E, Claesson-Welsh L (2016). Mechanisms and regulation of endothelial VEGF receptor signalling. *Nat Rev Mol Cell Biol*.

[48] Türker E, Garreis F, Khajavi N (2018). Vascular Endothelial Growth Factor (VEGF) Induced Downstream Responses to Transient Receptor Potential Vanilloid 1 (TRPV1) and 3-Iodothyronamine (3-T(1)AM) in Human Corneal Keratocytes. *Front Endocrinol (Lausanne)*.

[49] Thysen S, Luyten FP, Lories RJ (2015). Targets, models and challenges in osteoarthritis research. *Dis Model Mech*.

[50] Qiu S, Shi C, Anbazhagan AN (2020). Absence of VEGFR-1/Flt-1 signaling pathway in mice results in insensitivity to discogenic low back pain in an established disc injury mouse model. *J Cell Physiol*.

[51] Lin J, Li G, Den X (2010). VEGF and its receptor-2 involved in neuropathic pain transmission mediated by P2X₂(/)₃ receptor of primary sensory neurons. *Brain Res Bull*.

[52] Wang J, He C, Zhou T (2016). NGF increases VEGF expression and promotes cell proliferation via ERK1/2 and AKT signaling in Müller cells. *Mol Vis*.

[53] O-Sullivan I, Kc R, Singh G (2022). Sensory Neuron-Specific Deletion of Tropomyosin Receptor Kinase A (TrkA) in Mice Abolishes Osteoarthritis (OA) Pain via NGF/TrkA Intervention of Peripheral Sensitization. Int J Mol Sci.

[54] Nieto FR, Vuckovic SM, Prostran MS (2020). Editorial: Mechanisms and New Targets for the Treatment of Chronic Pain. *Front Pharmacol*.

[55] Van Wynsberghe M, Flejeo J, Sakhi H (2021). Nephrotoxicity of Anti-Angiogenic Therapies. *Diagnostics (Basel)*.

[56] Malyszko J, Kozlowska K, Kozlowski L (2017). Nephrotoxicity of anticancer treatment. *Nephrol Dial Transplant*.

[57] Sánchez-Gastaldo A, Kempf E, González Del Alba A (2017). Systemic treatment of renal cell cancer: A comprehensive review. *Cancer Treat Rev*.

