# Supplementary material for: Targeting Vascular Endothelial Growth Factor Receptors as a Therapeutic Strategy for Osteoarthritis and Associated Pain
Source: Int J Biol Sci. 2023 Jan 1;19(2):675–90. doi: 10.7150/ijbs.79125 (PMC9830519; doi:10.7150/ijbs.79125)
Supplement: Supplementary file 1 — Supplementary figures and tables. [file ijbsv19p0675s1.pdf]

Targeting Vascular Endothelial Growth Factor Receptors as a Therapeutic Strategy for Osteoarthritis and Associated Pain

Running title: Novel Targets for Osteoarthritis Treatment

Kaige Ma[1, 2], Gurjit Singh[1], Jun Wang[1], InSug O-Sullivan[1], Gina Votta-Velis[3], Benjamin Bruce[4], Arivarasu Natarajan Anbazhagan[5], Andre J. van Wijnen[1],[6*], Hee-Jeong Im[1, 4*]

AFFILIATIONS

Departments of [1]Biomedical Engineering, [3]Anesthesiology, and [5]Medicine, the University of Illinois at Chicago, Chicago, IL, USA

[2]Department of Orthopaedics, Union Hospital, Tongji Medical College, Huazhong University of Science and Technology, Wuhan, China

[4]Jesse Brown Veterans Affairs Medical Center (JBVAMC) at Chicago, IL 60612, USA

[6] Department of Biochemistry, University of Vermont, Burlington, VT 05405, USA

CORRESPONDING AUTHORS: * Hee-Jeong Im, Department of Biomedical Engineering, University of Illinois at Chicago, 851 S. Morgan St, Chicago, IL, USA. Email: hsampe2@uic.edu

* Dr. Andre J. van Wijnen, Department of Biochemistry, University of Vermont, Burlington, VT 05405, USA. E-mail address: andre.vanwijnen@uvm.edu

Supplementary Material

Supplementary figures and tables.

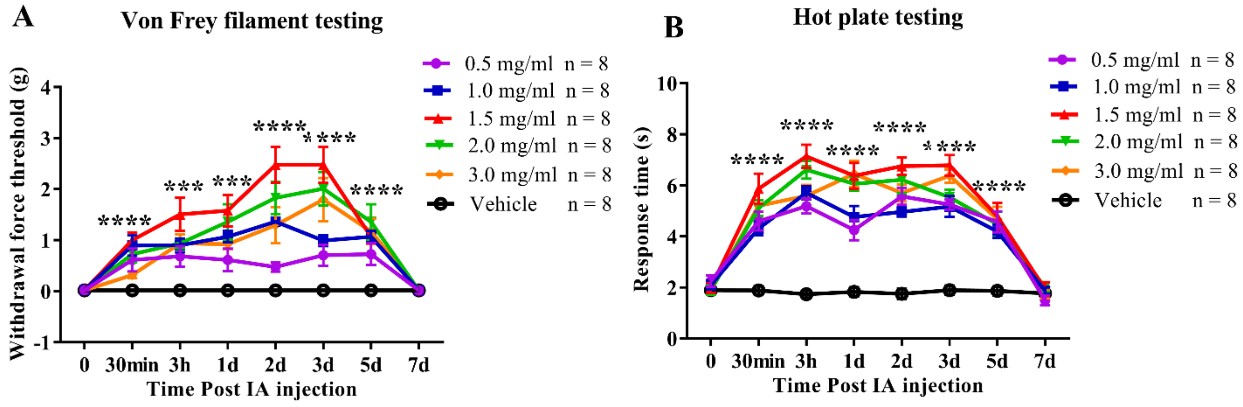

Figure S1. Dose-dependent pain response experiments. We determined the most effective concentrations and frequencies of drug treatments and drug schedules for pazopanib by concentration-dependent pain response experiments. Five different doses of pazopanib (5 µL of 0.5, 1, 1.5, 2 or 3 mg/mL/knee) were evaluated in female mice at week 4 (treatment for early OA) after partial medial meniscectomy (PMM). Von Frey (**A**) and hot plate (**B**) behavioral responses were measured after IA injection of pazopanib or vehicle (5% DMSO in PBS) in our surgery-induced OA murine model (n=8/group). Statistical analysis was conducted using one-way ANOVA with Tukey-Kramer test. Data are presented as mean ± SEM. ***$p<0.001$, ****$p<0.0001$ for comparisons between groups with or without pazopanib treatment (1.5mg/ml) in mice with PMM.

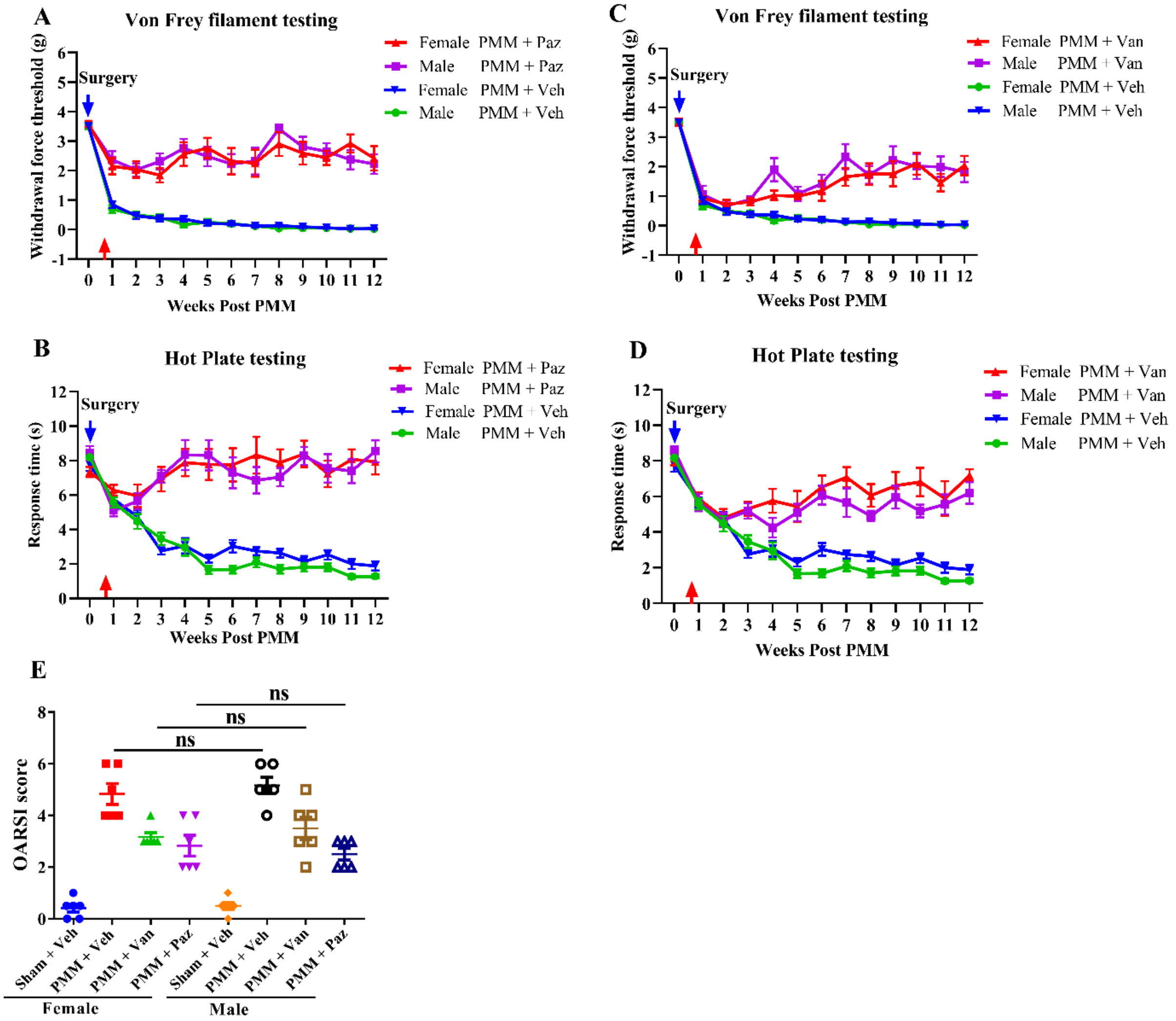

Figure S2. Sex differences in the chronic effects of pazopanib or vandetanib. There were no sex differences in the chronic effects of IA injection of pazopanib or vandetanib. Development of mechanical allodynia (von Frey filament testing) (**A**) and thermal pain assay (hot plate testing) (**B**) in the ipsilateral hind paw with IA injections of pazopanib (Paz) or vehicle (Veh, 5% DMSO in PBS) in male and female mice after partial medial meniscectomy (PMM) (female n=8, male n=10). Development of mechanical allodynia (von Frey filament testing) (**C**) and thermal pain assay (hot plate testing) (**D**) in the ipsilateral hind paw with IA injections of vandetanib (Van) or vehicle (Veh, 5% DMSO in PBS) in male and female mice after PMM (female

n=9, male n=9). Graphs of average OARSI scores of male and female mice (**E**) (n=6). Statistical analysis was conducted using one-way ANOVA with Tukey-Kramer test. Data are presented as mean ± SEM.

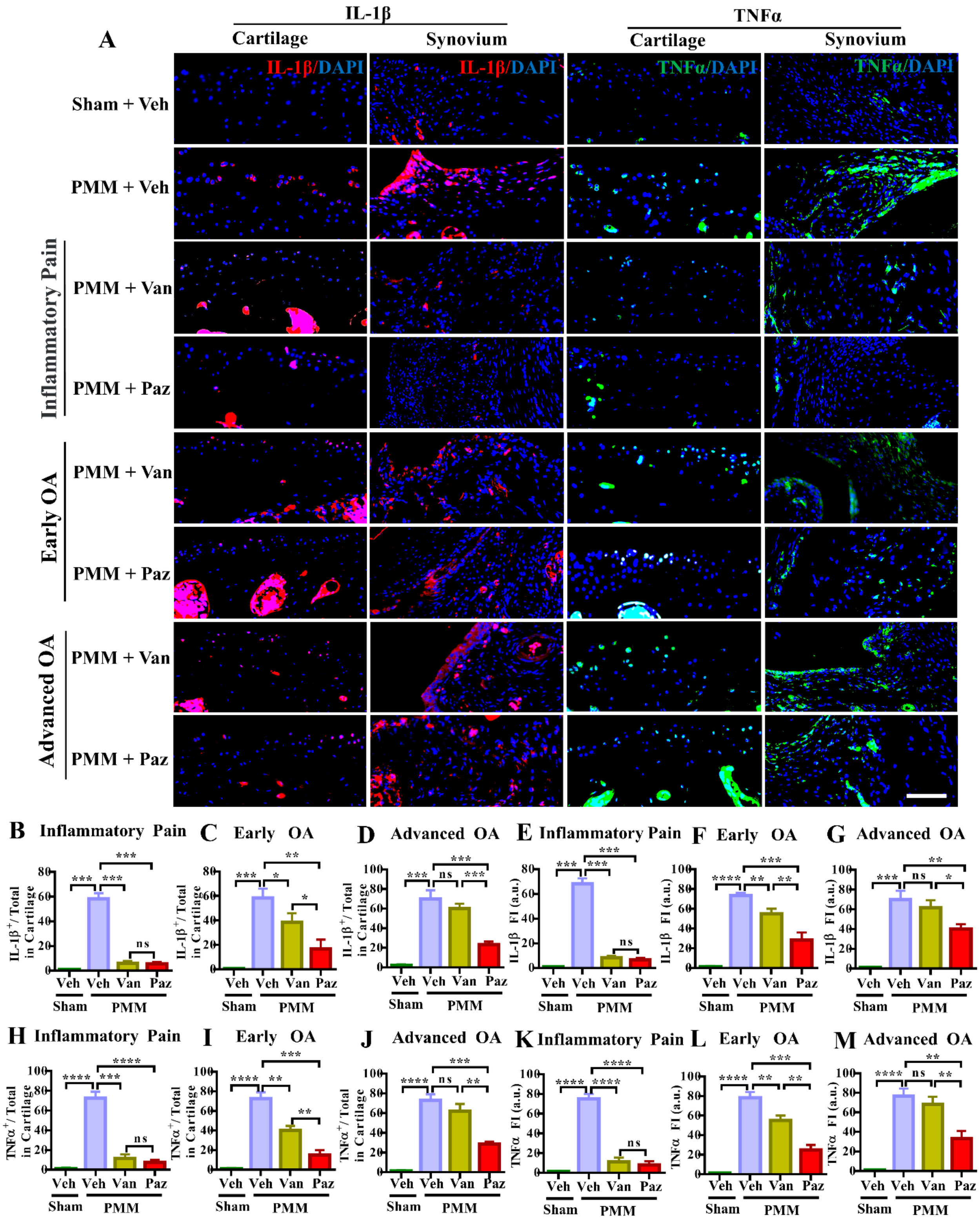

Figure S3. Inhibition of VEGFR1 or VEGFR2 decreases the expression of TNFα and IL-1β.

Inhibition of VEGFR1 or VEGFR2 is correlated with decreased expression of TNFα and IL-1β

during OA development and progression. Immunofluorescence (IF) assays were performed in histological sections of cartilage and synovial tissues in mice 12 weeks after partial medial meniscectomy (PMM). IA injection of pazopanib (Paz) or vandetanib (Van) or vehicle (Veh, 5% DMSO in PBS) was performed at week 1 (Gp1, inflammatory pain stage), week 4 (Gp2, early OA stage) or week 8 (Gp3, advanced OA stage) after PMM, twice per week for 12 weeks. The effects of drugs on the expression of IL-1β (red) and TNFα (green) in cartilage and synovium were examined by IF microscopy (**A**). Quantitative analysis of TNFα and IL-1β expression (n=3). Statistical analyses were conducted using one-way ANOVA with Tukey-Kramer test (**B-M**). Data are presented as mean ± SEM. *p<0.05, **p<0.01, ***p<0.001, ****p<0.0001 are for comparisons between groups with or without pazopanib or vandetanib treatment in mice with PMM. 4′,6-diamidino-2-phenylindole (DAPI) stains nuclei blue. Scale bars: 100 µm. FI, fluorescence intensity; a.u., arbitrary unit.

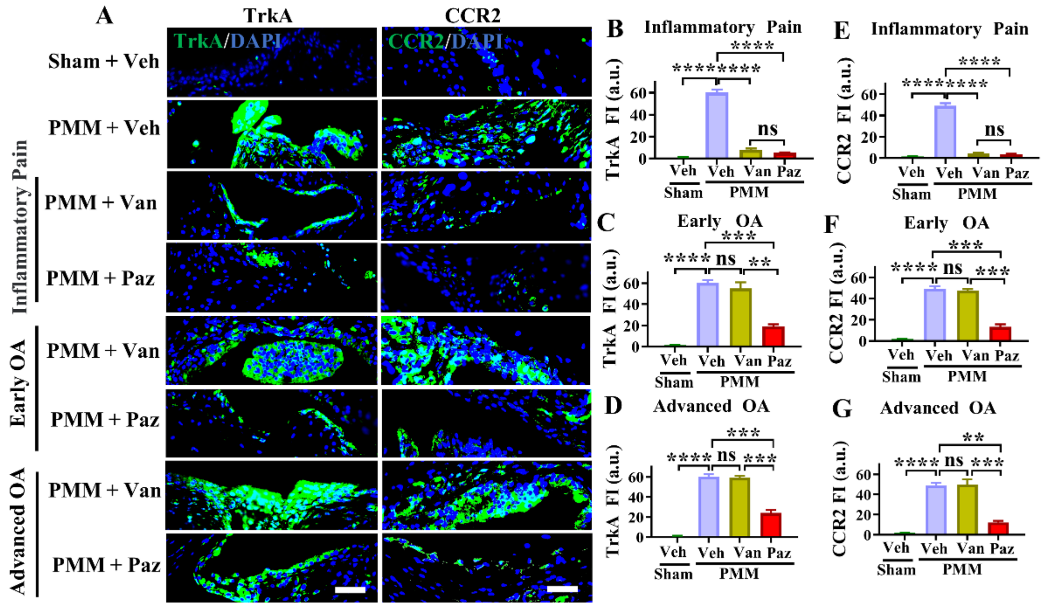

Figure S4. Pazopanib decreases expression of TrkA and CCR2 in synovium. Inhibition of VEGFR1 is correlated with decreased expression of TrkA during OA development and

progression. Immunofluorescence (IF) assays were performed in histological sections of synovial tissues in mice 12 weeks after partial medial meniscectomy (PMM). IA injection of pazopanib (Paz) or vandetanib (Van) or vehicle (Veh, 5% DMSO in PBS) was performed at week 1 (Gp1, inflammatory pain stage), week 4 (Gp2, early OA stage) or week 8 (Gp3, advanced OA stage) after PMM, twice per week for 12 weeks. The effects of drugs on the expression of TrkA and CCR2 (green) in synovium were examined by IF microscopy (**A**). Quantitative analysis of TrkA and CCR2 expression (n=3). Statistical analyses were conducted using one-way ANOVA with Tukey-Kramer test (**B-G**). Data are presented as mean ± SEM. **p<0.01, ***p<0.001, ****p<0.0001 are for comparisons between groups with or without pazopanib or vandetanib treatment in mice with PMM. 4′,6-diamidino-2-phenylindole (DAPI) stains nuclei blue. Scale bars: 100 μm. FI, fluorescence intensity; a.u., arbitrary unit.

79

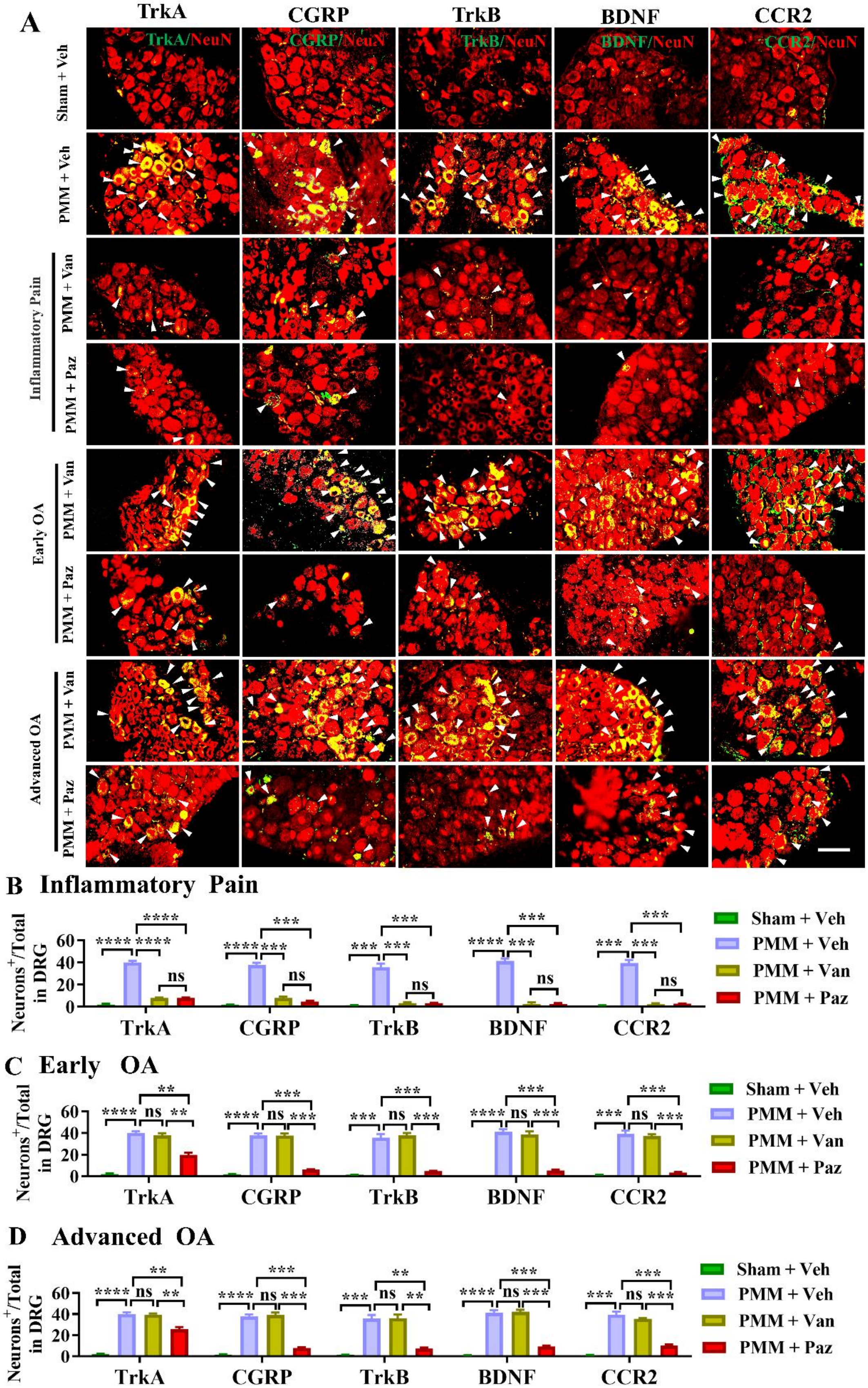
A
TrkA
CGRP
TrkB
BDNF
CCR2
TrkA/NeuN
CGRP/NeuN
TrkB/NeuN
BDNF/NeuN
CCR2/NeuN
Sham + Veh
PMM + Veh
Inflammatory Pain
PMM + Van
PMM + Paz
Early OA
Advanced OA
B Inflammatory Pain
C Early OA
D Advanced OA
Neurons+/Total in DRG
Sham + Veh
PMM + Veh
PMM + Van
PMM + Paz

Figure S5. Inhibition of VEGFR1 decreases expression of TrkA, CGRP, TrkB, BDNF and CCR2 in DRG sensory neurons during OA development and progression. Immunofluorescence (IF) assays were performed in histological sections of DRG tissues in mice 12 weeks after partial medial meniscectomy (PMM). IA injection of pazopanib (Paz) or vandetanib (Van) or vehicle (Veh, 5% DMSO in PBS) was done at week 1 (Gp1, inflammatory pain stage), week 4 (Gp2, early OA stage) or week 8 (Gp3, advanced OA stage) after PMM, twice per week for 12 weeks, and the drug effects on the expression of TrkA, CGRP, TrkB, BDNF and CCR2 in DRG were examined by dual immunostaining with NeuN (red), a neuronal marker, arrows indicate DRG neurons positive for TrkA, CGRP, TrkB, BDNF and CCR2 (green) (**A**). Quantitative analysis of TrkA, CGRP, TrkB, BDNF and CCR2 expression (n=3) (**B-D**). Statistical analyses were done using one-way ANOVA with Tukey-Kramer test. Data are presented as mean ± SEM. $^{**}p<0.01$, $^{***}p<0.001$, $^{****}p<0.0001$ are for comparisons between groups with or without pazopanib or vandetanib treatment in mice with PMM. Scale bars: 100 μm.

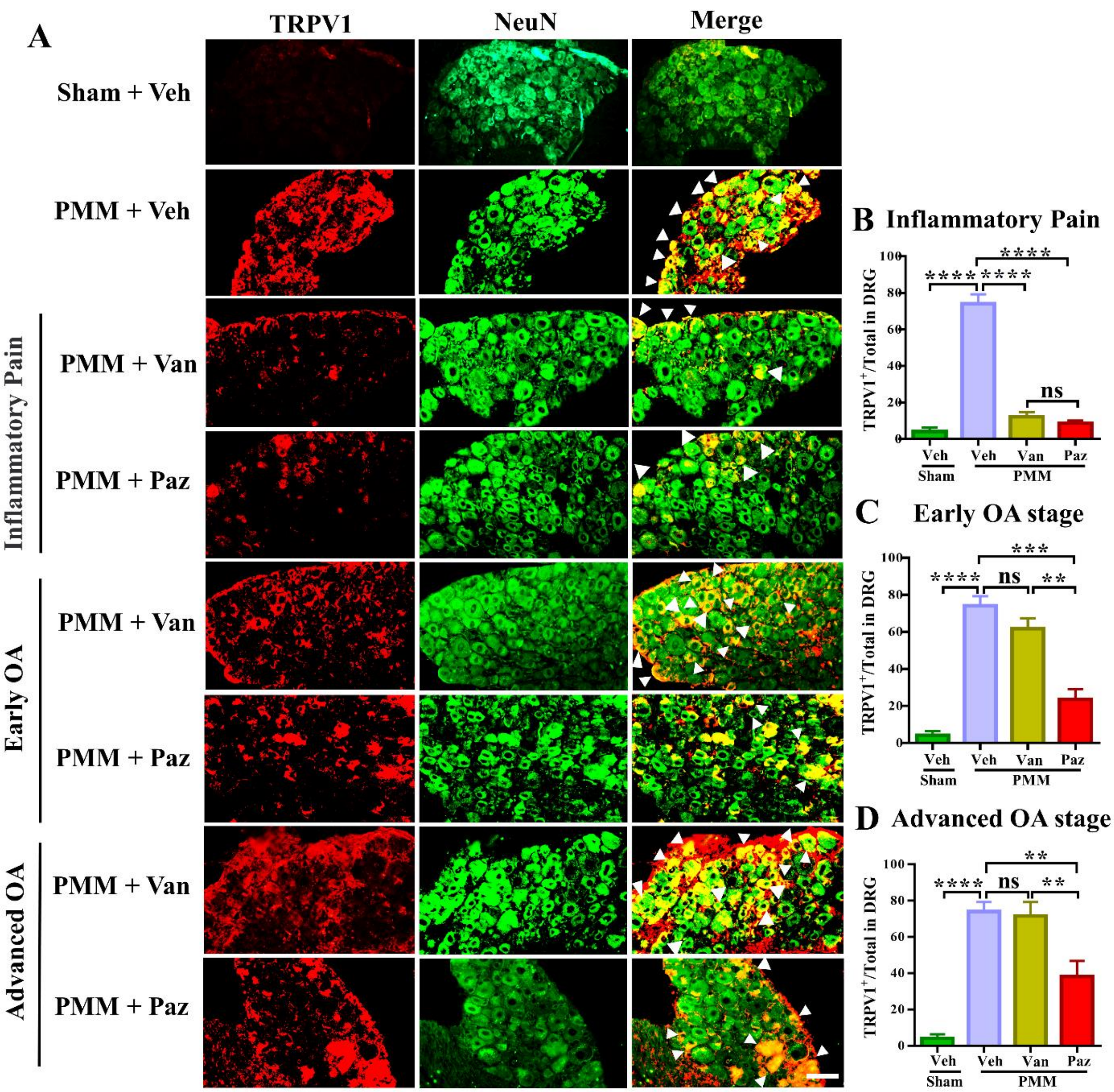

Figure S6. **Inhibition of VEGFR1 decreases the expression of TRPV1 in DRG sensory neurons during OA development and progression.** Immunofluorescence (IF) assays were performed in histological sections of DRG tissues in mice 12 weeks after partial medial meniscectomy (PMM). IA injection of pazopanib (Paz) or vandetanib (Van) or vehicle (Veh, 5% DMSO in PBS) was done at week 1 (Gp1, inflammatory pain stage), week 4 (Gp2, early OA stage) or week 8 (Gp3, advanced OA stage) after PMM, twice per week for 12 weeks, and the drug

effects on the expression of TRPV1 in DRG were examined by dual immunostaining with NeuN (green), a neuronal marker, arrows indicate DRG neurons positive for TRPV1 (red) (**A**). Quantitative analysis of TRPV1 expression (n=3) (**B-D**). Statistical analyses were done using one-way ANOVA with Tukey-Kramer test. Data are presented as mean ± SEM. **p<0.01, ***p<0.001, ****p<0.0001 are for comparisons between groups with or without pazopanib or vandetanib treatment in mice with PMM. Scale bars: 100 μm.

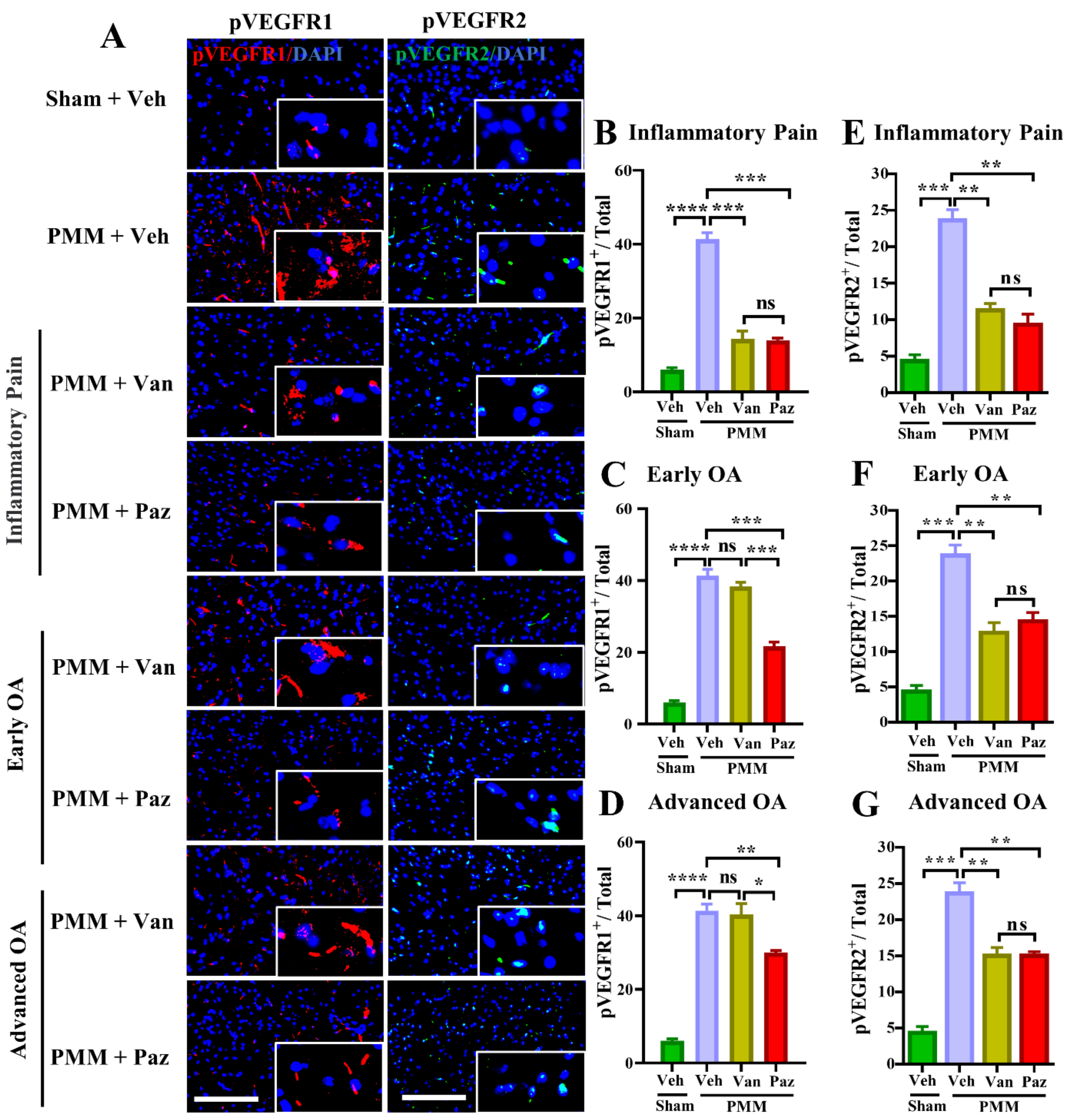

Figure S7. IA injection of Pazopanib inhibits activation of VEGFR1 in the dorsal horn of spinal cord. Immunofluorescence (IF) assays were done in histological sections of spinal cord tissues in mice 12 weeks after partial medial meniscectomy (PMM). IA injection of pazopanib (Paz) or vandetanib (Van) or vehicle (Veh, 5% DMSO in PBS) was done at week 1 (Gp1, inflammatory pain stage), week 4 (Gp2, early OA stage) or week 8 (Gp3, advanced OA stage) after PMM, twice per week for 12 weeks, and the drug effects on the altered activation of VEGFR1 (red) and VEGFR2 (green) in spinal cord were examined by IF microscopy (**A**). Quantitative analysis of pVEGFR1 and pVEGFR2 (n=3) (**B-G**). Statistical analyses were done using one-way ANOVA with Tukey-Kramer test. Data are presented as mean ± SEM. *$p<0.05$, **$p<0.01$, ***$p<0.001$, ****$p<0.0001$ for comparisons between groups with or without pazopanib or vandetanib treatment in mice with PMM. 4′,6-diamidino-2-phenylindole (DAPI) stains nuclei blue. Scale bars: 100 μm.

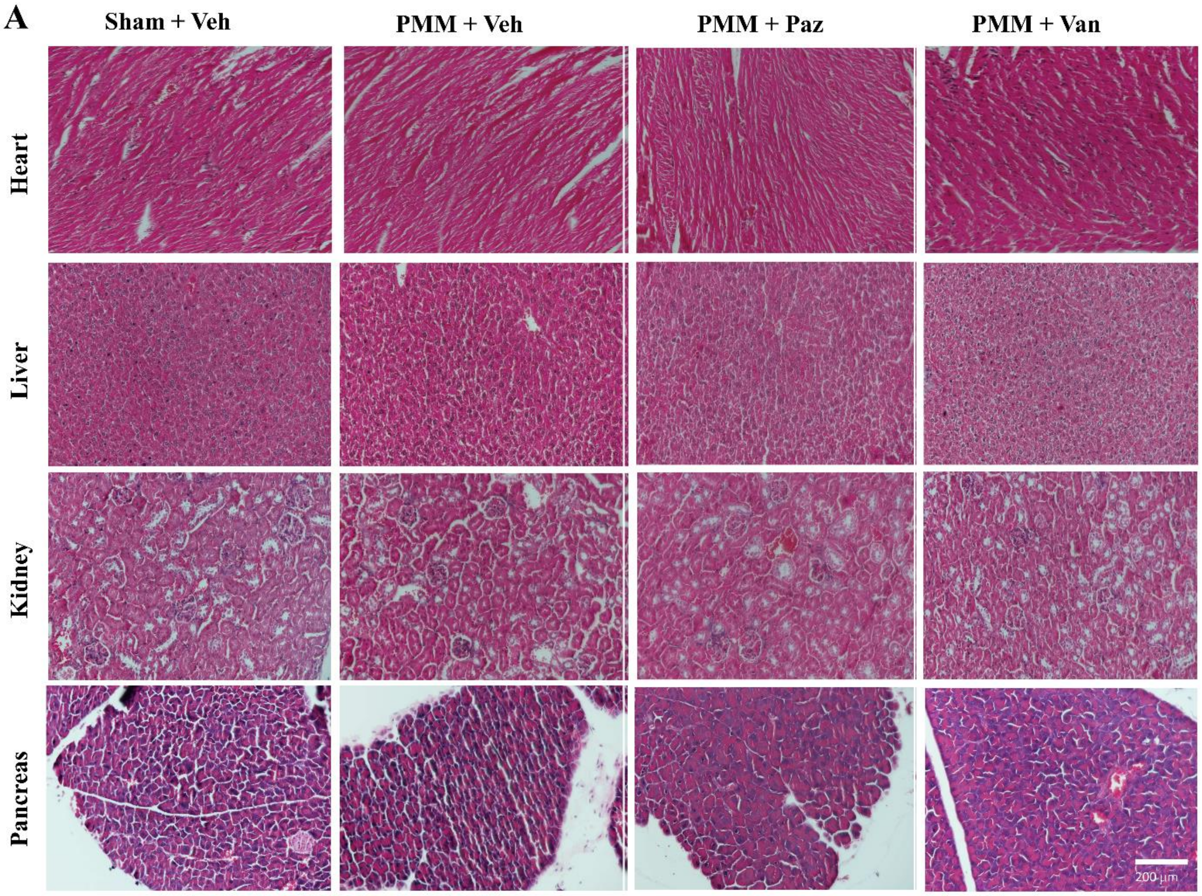

B

Organ	Changes	Sham + Veh	PMM + Veh	PMM + Paz	PMM + Van
Heart	Myocardial fibre degeneration	0/10	0/10	0/10	0/10
Liver	Granular degeneration, Fatty degeneration	0/10	0/10	0/10	0/10
Kidney	Granular degeneration	0/10	0/10	0/10	0/10
Pancreas	Pancreatic cell degeneration	0/10	0/10	0/10	0/10

Figure S8. Histopathological results of organs in our experimental murine OA model with/without chronic treatments of pazopanib or vandetanib for 12 weeks. IA injection (twice per week) of pazopanib (Paz) or vandetanib (Van) or vehicle (Veh, 5% DMSO in PBS) was done

at week 1 (Gp1, inflammatory pain stage) after partial medial meniscectomy (PMM). After drug/vehicle treatments for 12 weeks, the drug effects on the organs (heart, kidney, liver and pancreas) were examined by Hematoxylin & eosin (H&E) staining **(A)**. For a semi-quantitative comparison of the structural changes, the abnormalities in the tissue sections were graded from 0 (normal structure) to 3 (severe pathological changes). Statistical analyses were done using one-way ANOVA with Tukey-Kramer test **(B)**. Data are presented as mean ± SEM. Each organ tissue resulted in 10 images. No toxicity was observed using 10 images/organ. The toxicological score for all organ tissues was 0 (normal).

Table S1. Human real-time PCR primer sequences.

Gene	Forward primer	Reverse primer
VEGFR1	5’-GGCTGTTTTCTCTCGGATCTC-3’	5’-CATCTCCTCCGAGCCTGAAAG-3’
VEGFR2	5’-CTCAAGACAGGAAGACCAAGAA-3’	5’-GTCGTCTGATTCTCCAGGTTT-3’
TNFα	5’-ACCAGCTAAGAGGGAGAGAAGCAA-3’	5’-TCAGTGCTCATGGTGTCCTTTCCA-3’
RUNX2	5’-CCCAGTATGAGAGTAGGTGTCC-3’	5’-GGGTAAGACTGGTCATAGGACC-3’
COL10A1	5’-ACCCAAGGACTGGAATCTTTAC-3’	5’-GCCATTCTTATACAGGCCTACC-3’
MMP13	5’-CCTTGATGCCATTACCAGTCTCC-3’	5’-AAACAGCTCCGCATCAACCTGC-3’
ADAMTS5	5’-CTGTGACGGCATCATTGGCTCAAA-3’	5’-TTCAGGAATCCTCACCACGTCAGT-3’
SOX9	5’-TACTCCACCTTCACCTACATGAACCC-3’	5’-AAGGTCGAGTGAGCTGTGTGTAGA-3’
COL2A1	5’-AGAAGAACTGGTGGAGCAGCAAGA-3’	5-TGCTGTTCTTGCAGTGGTAGGTGA-3’
ACAN	5’-TCTTGGAGAAGGGAGTCCAACTCT-3’	5’-ACAGCTGCAGTGATGACCCTCAGA-3’
GAPDH	5’-TCGACAGTCAGCCGCATCTTCTTT-3’	5’-GCCCAATACGACCAAATCCGTTGA-3’

Table S2. Mouse real-time PCR primer sequences.

Gene	Forward primer	Reverse primer
Vegfr1	5’-TGGCCACCACTCAAGATTAC-3’	5’-TATAGACACCCTCATCCTCCTC-3’
Ngf	5’-TCCAATCCTGTTGAGAGTGG-3’	5’-CAGGCTGTGTCTATGCGGAT-3’
Trka	5’-CTCCGTCATGGCTGCTTT-3’	5’-AACAGCACATCAAGAGACCC-3’
Tnfα	5’-TATGAGCCCATATACCTGGGAGGA-3’	5’-TCCCTTCACAGAGCAATGACTCCA-3’
Il-1β	5’-TCGCTCAGGGTCACAAGAAA-3’	5’-ATCAGAGGCAAGGAGGAAACAC-3’
Ccl2	5’-ACTGCATCTGCCCTAAGGTCTTCA-3’	5’-AGAAGTGCTTGAGGTGGTTGTGGA-3’
Il-10	5’-CCAAGACCAAGGTGTCTACAA-3’	5’-GGAGTCCAGCAGACTCAATAC-3’
Il-18	5’-GGAGACCTGGAATCAGACAAC-3’	5’-CAGTCATATCCTCGAACACAGG-3’
Mmp13	5’-TCTTTATGGTCCAGGCGATGA-3’	5’-ATCAAGGGATAGGGCTGGGT-3’
Runx2	5’-GCCTTCAAGGTTGTAGCCCT-3’	5’-GTTCTCATCATTCCCGGCCA-3’
β-actin	5’-ACGATGCTCCCCGGGCTGTATT-3’	5’-TCTTGCTCTGGGCCTCGTCA3-3’
